# 2D Germanane‐Based Z‐Scheme Heterojunctions for Ultrafast Antibiotic Degradation and Mineralization

**DOI:** 10.1002/smsc.70375

**Published:** 2026-08-19

**Authors:** Yiming Lei, Xavier Sala, Jordi García‐Antón, Jose Muñoz

**Affiliations:** ^1^ Departament de Química Universitat Autònoma de Barcelona Cerdanyola del Vallès (Barcelona) Spain

**Keywords:** 2D semiconductors, antibiotics, germanane, photocatalysis, water remediation

## Abstract

The escalating global concern over antibiotic contamination in aquatic environments demands the development of efficient photocatalytic systems capable of achieving both rapid degradation and deep mineralization. Herein, an all‐solid‐state Z‐scheme heterojunction has been engineered through the rational wet‐chemical immobilization of tetrahedral Ag_3_PO_4_ nanocrystals onto 2D germanane (2D–GeH) nanosheets, with metallic Ag nanoparticles serving as a pivotal electron mediator. Under visible light irradiation, the resulting 2D–GeH/Ag/Ag_3_PO_4_ heterojunction exhibits ultrafast tetracycline (TC) degradation (96.1% in 3 min) together with deep mineralization efficiency (70.5% total organic carbon removal). Mechanistic investigations reveal the pivotal role of superoxide radicals in the degradation process, while chromatographic analyses identify two dominant TC transformation pathways leading to mineralization. Thus, this work highlights the potential of 2D–GeH‐based Z‐scheme heterojunctions as effective visible‐light‐driven photocatalysts for effective antibiotic remediation.

## Graphical abstract

An all‐solid‐state Z‐scheme 2D–GeH/Ag/Ag_3_PO_4_ heterojunction was engineered for visible‐light‐driven tetracycline removal, enabling ultrafast degradation (96.1% in 3 min) together with deep mineralization (70.5% TOC removal). These findings establish 2D Xene nanoarchitectonics as promising building blocks for advanced photocatalytic water remediation.

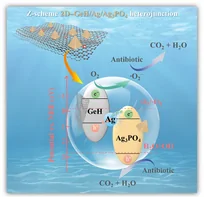

## 1 Introduction

The persistent presence of antibiotics in aquatic ecosystems has emerged as a critical environmental concern due to their potential to induce bacterial resistance and long‐term toxicity [1, 3]. Among the wide library of antibiotics, tetracycline (TC) stands out as one of the most extensively abused broad‐spectrum antibiotics [4, 5]. Briefly, TC is notorious for its chemical stability and low metabolic rate, leading to its ubiquitous presence in surface waters and the subsequent proliferation of antibiotic‐resistant bacteria [6]. These concerns, together with the potential formation of by‐products that may exhibit even greater toxicity, underscore the urgent need for developing advanced oxidation systems capable not only of degrading antibiotics into intermediate products, but also of achieving deep mineralization into innocuous inorganic species, such as CO_2_ and H_2_O.

While semiconductor photocatalysis offers an appealing solution for antibiotics remediation [7, 9], traditional semiconductors (e.g., TiO_2_ [10], ZnO [11], WO_3_ [12], BiVO_4_ [13], MoS_2_ [14], Ag_3_PO_4_ [15]) are often hindered by inefficient visible‐light absorption and rapid electron–hole (e^–^–h^+^) recombination rates [16, 19], which results in incomplete degradation and the accumulation of toxic intermediates [20]. Inspired by the stepwise photon excitation process of natural photosynthesis, the Materials Chemistry community has moved to engineering Z‐scheme heterojunctions to promote advanced oxidation processes (AOPs) [21, 23]. In such systems, two semiconductors are combined to sustain spatial charge separation while preserving strong redox potentials for water splitting [24]. In addition, an electron mediator (e.g., metal nanoparticles (NPs), such as Ag‐NPs or Au‐NPs) is commonly incorporated into the heterojunction to maintain high‐energy charge carriers needed to drive pollutant degradation [25].

Beyond the paradigm of graphene, 2D Xenes—specifically 2D germanane (2D–GeH)—have emerged as a growing class of 2D semiconductors with exceptional structural, electronic, optical, and quantum properties [26, 28], making them suitable for a wide range of applications [29, 32]. Unlike graphene, which exhibits a zero‐bandgap semiminetallic nature, 2D–GeH possesses a direct and tunable bandgap located in the visible region [33, 34]. Despite these advantages, the valence band position of 2D–GeH is insufficiently positive to trigger the direct oxidation of water into hydroxyl radicals (•OH), thereby limiting its overall oxidative capacity in environmental remediation. Although promising theoretical results support 2D–GeH as promising photocatalysts for Z‐scheme heterojunction development [35, 37], its real implementation is nowadays yet to be experimentally validated.

**SCHEME 1:**
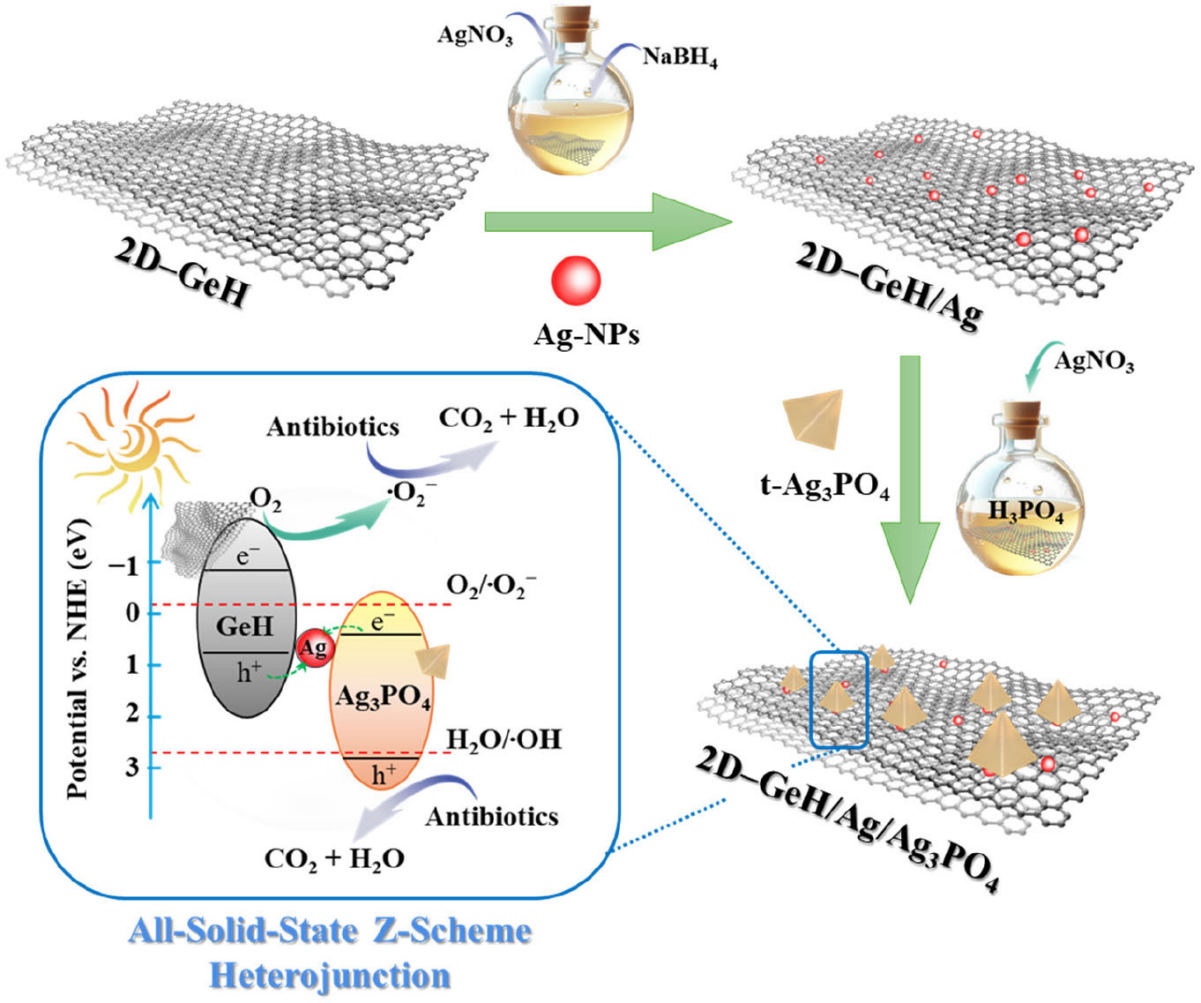
Synthetic illustration for all‐solid‐state Z‐scheme 2D–GeH/Ag/Ag_3_PO_4_ heterojunction for TC degradation and mineralization. 2D–GeH nanosheets were functionalized with Ag‐NPs followed by t‐Ag_3_PO_4_ crystallization via a wet‐chemical approach. Inset: All‐solid‐state Z‐scheme charge–transfer mechanism in 2D–GeH/Ag/Ag_3_PO_4_ heterojunction for TC degradation.

Herein, we report the rational design of a 2D–GeH‐based all‐solid‐state Z‐scheme heterojunction for photocatalytic antibiotic degradation and mineralization, using TC as a model target. For this goal, 2D–GeH nanosheets have been functionalized with both Ag‐NPs and facet‐controlled tetrahedral silver phosphate (t‐Ag_3_PO_4_) nanocrystals via a wet‐chemical assembly approach [38, 39], resulting in the 2D–GeH/Ag/Ag_3_PO_4_ heterojunction (see Scheme 1 for illustration). This architecture not only facilitates spatial charge separation but also preserves the high‐energy redox states of the individual semiconductors. After an accurate characterization, the optimized 2D–GeH/Ag/Ag_3_PO_4_ heterojunction demonstrated ultrafast TC removal (96.1% in 3 min) and deep mineralization efficiency (70.5% total organic carbon removal, TOC) under visible light irradiation, while promising results were also achieved under simulated sunlight irradiation. Finally, comprehensive spectroscopic, electrochemical, and chromatographic analyses elucidated the Z‐scheme charge–transfer mechanism and the transformation pathways of TC. All in all, this work establishes 2D–GeH as a promising AOP platform for sunlight‐driven environmental remediation applications.

## 2 Experimental Section

### 2.1 Chemicals

2D germanane (2D–GeH), silver nitrate (AgNO_3_), sodium monohydrogen phosphate (Na_2_HPO_4_), phosphoric acid (H_3_PO_4_), *t*‐butyl alcohol (TBA), methanol (MeOH), ethanol (EtOH), tetracycline (TC), ciprofloxacin (CIP), and sodium sulfate (Na_2_SO_4_) were purchased from Sigma–Aldrich (St. Louis, MO, USA). Argon (Ar) gas was purchased from Air Liquide (Madrid, Spain).

### 2.2 Functionalization of 2D–GeH With Ag‐NPs

Fifteen milliliters of a 1 mg·mL^−1^ aqueous dispersion of 2D–GeH were mixed with 250 μL of a 1 mg·mL^−1^ AgNO_3_ aqueous solution. The mixture was stirred for 1 h to allow efficient adsorption of Ag^+^ onto the 2D–GeH surface. Subsequently, 100 mg of NaBH_4_ was added to the mixture and stirred for 3 h to induce in situ Ag‐NPs formation via Ag^+^ reduction. All the steps were carried out under an Ar atmosphere to prevent 2D–GeH oxidation. Finally, the resulting 2D–GeH/Ag material was washed several times with deionized water and collected by centrifugation.

### 2.3 Synthesis of 2D–GeH/Ag/Ag_3_PO_4_ Heterojunction

The tetrahedral‐facet 2D–GeH/Ag/Ag_3_PO_4_ heterojunction was prepared by the dropwise addition of 6.44 mL of a 0.1 M AgNO_3_ EtOH solution to 10 mL of a 0.1 M H_3_PO_4_ EtOH solution containing 5 mg of 2D–GeH/Ag [39, 40]. After continuous stirring for 60 min at 60 °C, the resulting 2D–GeH/Ag/Ag_3_PO_4_ heterojunction (2D–GeH/Ag:Ag_3_PO_4_ = 2:8) was collected by centrifugation, washed several times with deionized water, and dried under vacuum to avoid the oxidation of 2D–GeH/Ag. To optimize the ratio between 2D–GeH/Ag and Ag_3_PO_4_, different amounts of 0.1 M AgNO_3_ in EtOH solution (from 0.4 to 1.6 mL) were dropped into the same 2D–GeH/Ag dispersion.

For comparison, an amorphous heterojunction (viz. 2D–GeH/Ag/a‐Ag_3_PO_4_) was synthesized by dispersing 5 mg of 2D–GeH in 10 mL of 0.08 M AgNO_3_ aqueous solution under vigorous stirring for 60 min, followed by the dropwise addition of 1.8 mL of a 0.15 M Na_2_HPO_4_ aqueous solution. The a‐2D–GeH/Ag/Ag_3_PO_4_ material (2D–GeH/Ag:Ag_3_PO_4_ = 2:8) was centrifuged, washed with deionized water, and dried under vacuum to avoid the oxidation of 2D–GeH/Ag.

Both isolated amorphous (a‐Ag_3_PO_4_) and tetrahedral (t‐Ag_3_PO_4_) reference samples were synthesized following the same procedures in the absence of 2D–GeH/Ag.

Finally, a physically mixed control sample―denoted as Mix 2D–GeH/Ag···t‐Ag_3_PO_4_―was prepared by dispersing presynthesized 2D–GeH/Ag and t‐Ag_3_PO_4_ at the same weight ratio used for the chemically assembled 2D–GeH/Ag/Ag_3_PO_4_ heterojunction (2:8). The suspension was magnetically stirred for 1 h at room temperature, collected by centrifugation, and subsequently used in photocatalytic degradation experiments.

### 2.4 Apparatus and Procedures

The phase structure of the materials was characterized by powder X‐ray diffraction (XRD), using a PANalytical X’Pert Pro Powder Diffraction with Cu K_α_ radiation and a PIXcel1D detector. The morphology was studied by Transmission Electron Microscopy (TEM), using a JEM‐2011 unit with an acceleration voltage of 200 kV. Scanning electron microscopy with energy‐dispersive X‐ray (SEM‐EDX) spectroscopy was carried out on Zeiss Merlin. XPS characterizations were completed by a SPECS PHOIBOS 150 hemispherical analyzer (SPECS GmbH, Berlin, Germany). Ultraviolet–Visible (UV–vis) diffuse reflection spectroscopy (DRS) was performed using a V‐730 JASCO spectrophotometer. The total organic carbon (TOC) removal ratio was analyzed by a Shimadzu TOCVCPH analyzer based on High‐Temperature Combustion. After 3 min of photocatalytic reaction, the suspension was filtered through a cellulose acetate syringe filter, and the resulting supernatant was subsequently analyzed. Photoluminescence (PL) spectra were acquired using a Cary Eclipse Fluorimeter.

The identification of degradation intermediates was performed using a high‐performance liquid chromatography–mass spectrometry (HPLC‐MS) system (Agilent 1290 II HPLC coupled with an Agilent 6460 mass spectrometer) operating in electrospray ionization (ESI) mode. During the TC degradation experiments, aliquots of the reaction suspension were withdrawn after 3 min of photocatalytic reaction for intermediate identification. Chromatographic separation was carried out on a BEH‐C18 column (100 mm × 2.1 mm, 1.7 μm) maintained at 40 °C. The mobile phase consisted of acetonitrile and 0.1% formic acid (25:75, v/v) at a flow rate of 0.4 mL·min^−1^ and an injection volume of 10 μL. The instrumental parameters were set at a fragmentor voltage of 100 V, collision energy offset of 0, and cell exit potential offset of 0.

### 2.5 Photocatalytic TC Degradation

The photocatalytic activity of 2D–GeH/Ag/Ag_3_PO_4_ heterojunction was evaluated by using TC broad‐spectrum antibiotic as the model target. Firstly, a 0.2 mg·mL^−1^ dispersion of 2D–GeH/Ag/Ag_3_PO_4_ was mixed with 20 mg·L^−1^ (i.e., 20 ppm) TC solution and left aging in dark conditions for 25 min to achieve the adsorption–desorption equilibrium. Then, a LED lamp with an emission wavelength (λ) from 420 to 780 nm was used as the light source for inducing a photocatalytic degradation reaction (light intensity was calibrated to 1 sun). The concentration of TC was monitored by the area under the curve (Auc) of the characteristic absorption peak centered at ~357 nm in the UV‐Vis spectra [41]. The degradation efficiency (*η*) of TC was calculated as follows

$$\eta (\%)=\frac{\left(C_{0}-C_{t}\right)}{C_{0}}\times 100$$

where *C*_0_ and *C* *_t_* are the TC concentrations at time 0 and time *t* (min) after light illumination, respectively. Therefore, *C*_0_ corresponds to the TC concentration after the adsorption–desorption equilibrium was achieved under dark conditions.

The reaction kinetics and reaction rate constants were determined by the pseudo‐second‐order model, in which *k*_2_ corresponds to the pseudo‐second‐order reaction rate constant

$$\text{−}\frac{dC_{t}}{dt}=k_{2}\cdot C_{t}^{2}$$

Realistic conditions were performed by conducting the experiments with a solar simulator (LCS‐100, Oriel, UK, light intensity: 1 sun), while tap water sample was obtained from the Autonomous University of Barcelona (Spain).

### 2.6 Photoelectrochemical Measurements

Photoelectrochemical (PEC) measurements were performed by drop‐casting 100 μL of each tested sample (5 mg·mL^−1^) onto fluorine‐doped tin oxide (FTO) electrodes, which served as photoanodes. The PEC experiments were conducted in a conventional three‐electrode quartz cell using a PalmSens4 potentiostat/galvanostat equipped with the PSTrace software. A Pt wire, an Ag/AgCl (sat. KCl) electrode, and the prepared photoanodes were employed as the counter, reference, and working electrodes, respectively. A 1 M Na_2_SO_4_ aqueous solution was utilized as an electrolyte. Electrochemical impedance spectroscopy (EIS) measurements were recorded under LED irradiation. Nyquist plots were obtained at open‐circuit potential, whereas Mott–Schottky (M‐S) analyses were performed under an applied bias from 0.2 to 1.0 V (vs. NHE).

## 3 Results and Discussion

### 3.1 Synthesis and Characterization of 2D–GeH/Ag/Ag_3_PO_4_ Heterojunction

**FIGURE 1:**
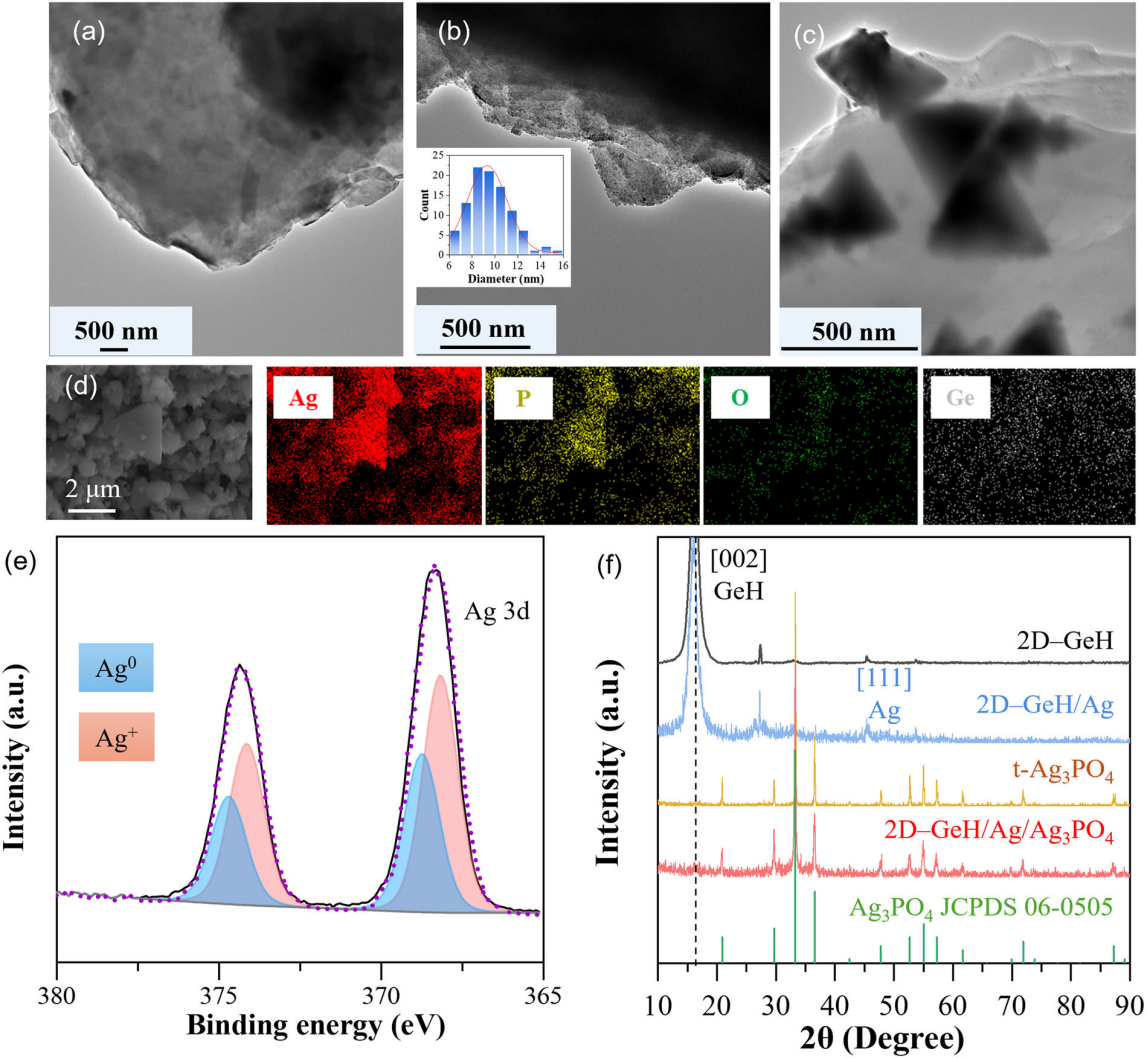
Material characterization of 2D–GeH/Ag/Ag_3_PO_4_ heterojunction. TEM images of (a) pristine 2D–GeH, (b) 2D–GeH, and (c) 2D–GeH/Ag/Ag_3_PO_4_. (d) EDX elemental mapping of the 2D–GeH/Ag/Ag_3_PO_4_ heterojunction for Ag, P, O, and Ge elements. (e) High‐resolution XPS spectrum of the Ag 3d core‐level for 2D–GeH/Ag/Ag_3_PO_4_ heterojunction. (f) XRD patterns of pristine 2D–GeH, 2D–GeH/Ag, t‐Ag_3_PO_4_, and 2D–GeH/Ag/Ag_3_PO_4_.

The 2D–GeH/Ag/Ag_3_PO_4_ heterojunction was rationally engineered by immobilizing both Ag‐NPs and tetrahedral Ag_3_PO_4_ (t‐Ag_3_PO_4_) onto 2D–GeH nanosheets through a wet‐chemical approach (see Scheme 1). Briefly, Ag‐NPs were crystallized onto 2D–GeH by mixing the 2D Xene with the Ag precursor (AgNO_3_), followed by reduction with NaBH_4_. Then, the resulting 2D–GeH/Ag was utilized as a nanotemplate for immobilization of tetrahedral‐faced Ag_3_PO_4_ nanocrystals, leading to the 2D–GeH/Ag/Ag_3_PO_4_ heterojunction. The ratio between 2D–GeH/Ag and Ag_3_PO_4_ was modulated to optimize the 2D–GeH/Ag/Ag_3_PO_4_ heterojunction. The resulting 2D Xene‐based heterojunction was comprehensively verified by a set of characterization techniques, including TEM, EDX, XPS, and XRD (see Figure 1).

The morphology of 2D–GeH at each functionalization stage was investigated by TEM images. Figure 1a shows the expected 2D multilayer structure of pristine 2D–GeH [27]. After Ag‐NPs incorporation, the 2D–GeH/Ag heterostructure presented a homogeneous distribution of spherical Ag‐NPs, with an average size of 9 ± 3 nm (Figure 1b). As shown in Figure 1c, the TEM micrograph of the 2D–GeH/Ag/Ag_3_PO_4_ heterojunction revealed the formation of well‐defined tetrahedral Ag_3_PO_4_ nanocrystals, whose morphology closely matches that of the isolated t‐Ag_3_PO_4_ reference (Figure S1). This indicates that their nucleation and growth proceed without detrimental distortion on the 2D scaffold. The EDX elemental mapping image of the 2D–GeH/Ag/Ag_3_PO_4_ heterojunction (Figure 1d) confirmed the spatial colocalization of Ag, P, and O through the Ge‐based 2D nanosheets. In addition, the high‐resolution XPS spectrum of Ag 3d verified the coexistence of both Ag^0^ (368.7 and 374.7 eV) and Ag^+^ (368.1 and 374.1 eV) species in the 2D–GeH/Ag/Ag_3_PO_4_ heterojunction (see Figure 1e), in line with the simultaneous presence of Ag‐NPs and t‐Ag_3_PO_4_. Table S1 summarized the elemental composition (Ge, Ag, P, and O), estimated from the corresponding peak areas. Based on these results, loading of Ag‐NPs and t‐Ag_3_PO_4_ on the 2D Xene was calculated to be 10.6% and 15.9%, respectively.

The structural integrity of the individual and hybrid materials was assessed by powder XRD (Figure 1f). The XRD patterns of pristine 2D–GeH displayed characteristic reflections at 16.2º and 27.3º, assigned to [002] and [100] planes, respectively [30, 42]. After functionalization with Ag‐NPs, the 2D–GeH/Ag pattern presented the main [111] plane for Ag at 38.3º, whose low intensity might be ascribed to the small crystallite size and modest loading of Ag‐NPs. Upon t‐Ag_3_PO_4_ incorporation, the resulting 2D–GeH/Ag/Ag_3_PO_4_ heterojunction presented a set of additional peaks which perfectly match with the body‐centered cubic crystalline structure of Ag_3_PO_4_ (6.004 Å, BCC, JCPDS no. 06–0505) [39, 43]. Importantly, the positions and relative intensities of the {200} and {222} reflections—centered at 29.7º and 52.7º, respectively—closely mirror those of the isolated t‐Ag_3_PO_4_ sample. The relative intensity ratio of the {222} and {200} diffraction planes around 0.92 evidences a dominant exposure of the {111} facets, indicating that the tetrahedral phase and crystallinity are preserved in the 2D–GeH/Ag/Ag_3_PO_4_ heterojunction [44].

Overall, this material characterization demonstrates the successful synthesis of the 2D–GeH/Ag/Ag_3_PO_4_ heterojunction via a wet‐chemical functionalization approach.

### 3.2 Photocatalytic Performance Towards TC Degradation

The photocatalytic activity of the 2D–GeH/Ag/Ag_3_PO_4_ heterojunction for antibiotic degradation was evaluated under visible‐light irradiation with a LED lamp (wavelength: 420–780 nm; light intensity: 1 sun), whereas tetracycline (TC) was selected as a model antibiotic target for the advanced oxidation process (AOP). For comparison, control experiments were conducted employing pristine 2D–GeH, 2D–GeH/Ag, and isolated t‐Ag_3_PO_4_ nanocrystals.

**FIGURE 2:**
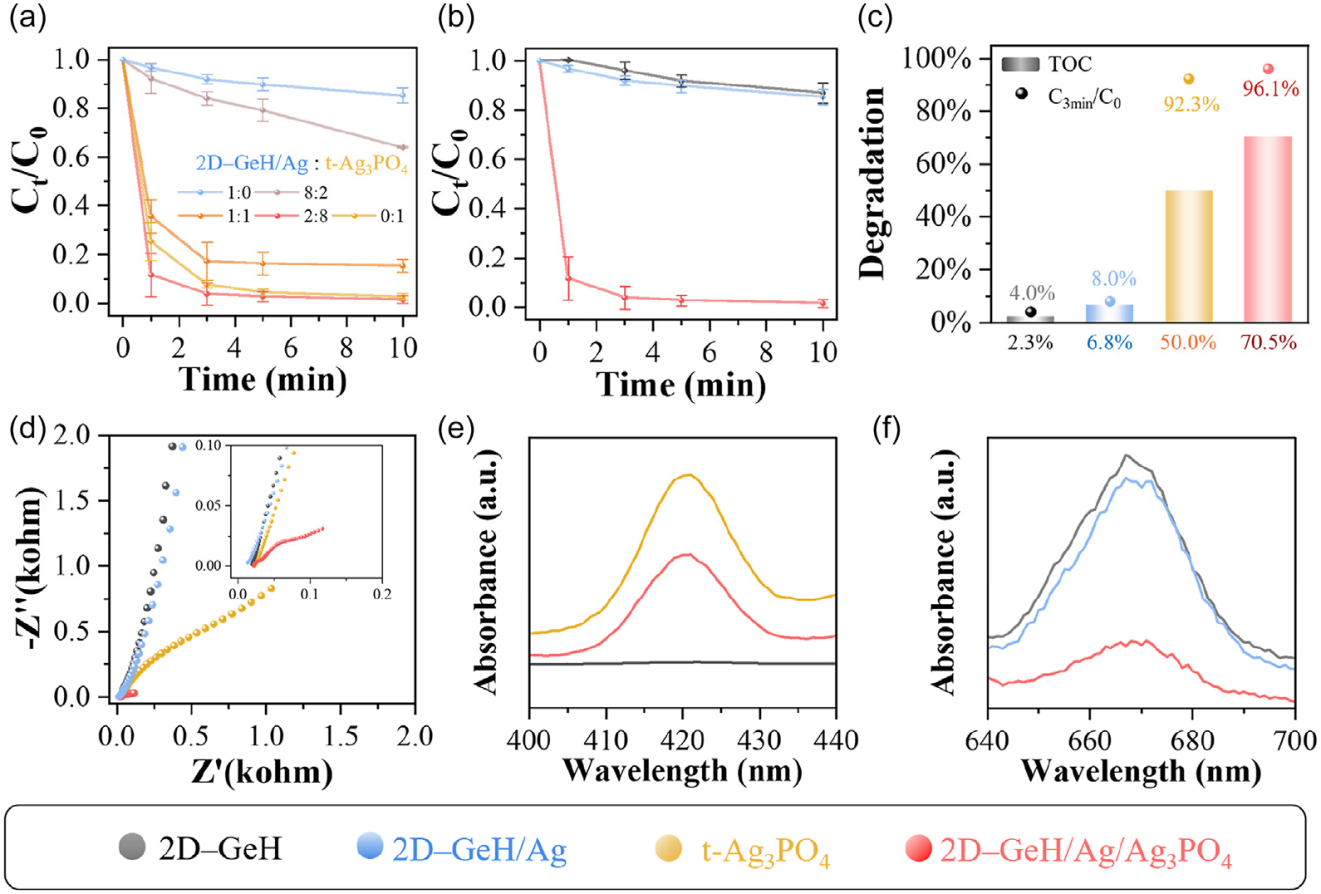
Photocatalytic degradation efficiency and reaction dynamics characterizations. Time‐dependent photocatalytic degradation of TC over (a) 2D–GeH/Ag/Ag_3_PO_4_ at different 2D–GeH/Ag:t‐Ag_3_PO_4_ molar ratios from 0:1 to 1:0; and (b) pristine 2D–GeH, 2D–GeH/Ag, and 2D–GeH/Ag/Ag_3_PO_4_ heterojunction. Reaction conditions: initial TC concentration: 20 mg·L^−1^; material dispersion: 0.2 mg·mL^−1^, light source: LED lamp with visible light (wavelength: 420–780 nm; light intensity: 1 sun). (c) Comparison in TC mineralization (column) and degradation (dots) efficiency of pristine 2D–GeH, 2D–GeH/Ag, t‐Ag_3_PO_4_, and 2D–GeH/Ag/Ag_3_PO_4_ after irradiation by visible light for 3 min. (d) Nyquist plots of pristine 2D–GeH, 2D–GeH/Ag, t‐Ag_3_PO_4_, and 2D–GeH/Ag/Ag_3_PO_4_. PL spectroscopy of (e) 2D–GeH, t‐Ag_3_PO_4_, and 2D–GeH/Ag/Ag_3_PO_4_ (excitation wavelength: 250 nm) and (f) pristine 2D–GeH, 2D–GeH/Ag, and 2D–GeH/Ag/Ag_3_PO_4_ (excitation wavelength: 500 nm).

First, the composition of the 2D–GeH/Ag/Ag_3_PO_4_ heterojunction for TC photodegradation was optimized by modulating the 2D–GeH/Ag:t‐Ag_3_PO_4_ ratio. As shown in Figure 2a, the optimum 2D–GeH/Ag:t‐Ag_3_PO_4_ ratio was found to be 2:8. Consequently, this composition was used for subsequent performance analyses. Figure S2 presents the UV–vis spectra resulting from the photodegradation rates of 20 mg·L^−1^ TC at different irradiation times in the absence (blank) and presence of 0.2 mg·mL^−1^ of 2D–GeH/Ag/Ag_3_PO_4_ heterojunction. While the 2D–GeH/Ag/Ag_3_PO_4_ heterojunction exhibited outstanding photodegradation activity for TC during visible irradiation exposure (96.1% in just 3 min), self‐degradation of TC was found to be negligible after 30 min. Table S2 presents a comparison of the photocatalytic efficiency of the 2D–GeH/Ag/Ag_3_PO_4_ heterojunction with that of recently reported photocatalysts for TC degradation.

Next, the time‐dependent photocatalytic degradation plots of Figure 2b demonstrated that control materials (viz. pristine 2D–GeH and 2D–GeH/Ag) exhibited insignificant photocatalytic AOP abilities, with a degradation efficiency (*η*) at 3 min as low as 4.0% and 8.0%, respectively. In contrast, isolated t‐Ag_3_PO_4_ nanocrystals presented an excellent *η* of 92.3%, which is close to that obtained by the 2D–GeH/Ag/Ag_3_PO_4_ heterojunction (96.1%). These results point out the ultrafast photocatalytic activity of the 2D–GeH/Ag/Ag_3_PO_4_ heterojunction, while evidencing the key role of t‐Ag_3_PO_4_ in TC degradation. Quantification values are summarized in Table S3. Kinetic analysis revealed that the TC photodegradation process over all samples was well described by a pseudo‐second‐order kinetic model (Figure S3). The resulting second‐order rate constants (*k*_2_) confirmed the superior photocatalytic activity of the 2D–GeH/Ag/Ag_3_PO_4_ heterojunction (*k*_2_ = 6.3 L⋅mg^−1^⋅min^−1^), outperforming pristine 2D–GeH (0.02 L⋅mg^−1^⋅min^−1^), 2D–GeH/Ag (0.02 L⋅mg^−1^⋅min^−1^), and isolated t‐Ag_3_PO_4_ nanocrystals (3.8 L⋅mg^−1^⋅min^−1^).

The recyclability of the 2D–GeH/Ag/Ag_3_PO_4_ heterojunction for TC degradation was also evaluated by carrying out cycling stability tests (Figure S4). Importantly, a negligible decrease in degradation efficiency was observed after three consecutive photocatalytic cycles, demonstrating its excellent operational stability and reusability.

Since during photocatalytic degradation, numerous intermediate compounds can be formed—and some of them can be even more hazardous than the initial pollutant—the mineralization ability of photocatalysts to provide nontoxic and harmless by‐products is also a pivotal indicator for evaluating their performances. Figure 2c columns illustrate the mineralization of TC under visible light irradiation, which was quantified by TOC removal after 3 min. The 2D–GeH/Ag/Ag_3_PO_4_ heterojunction yielded an excellent 70.5% TOC removal, which was markedly higher than that achieved by control materials: pristine 2D–GeH (2.3%), 2D–GeH/Ag (6.8%), and isolated t‐Ag_3_PO_4_ nanocrystals (50.0%). These results indicated that TC is not merely transformed into smaller fragments but is effectively mineralized into CO_2_ and H_2_O, thereby demonstrating an overall greener AOP profile for the 2D–GeH/Ag/Ag_3_PO_4_ heterojunction.

To further demonstrate the practical applicability of the as‐developed 2D–GeH/Ag/Ag_3_PO_4_ heterojunction, its photocatalytic degradation performance was evaluated using a solar simulator. Under simulated sunlight irradiation, the 2D–GeH/Ag/Ag_3_PO_4_ heterojunction displayed excellent TC degradation efficiency (91.6%) in only 3 min (vs. 96.1% under visible light irradiation; see Figure S5). These results point out the strong potential of 2D Xene‐based heterojunctions for efficient photocatalytic pollutant remediation under realistic environmental conditions.

Lastly, the impact of the chemically assembled 2D–GeH/Ag/Ag_3_PO_4_ heterojunction on photocatalytic TC degradation was assessed by comparison with a physically mixed control sample (viz. Mix 2D–GeH/Ag···t‐Ag_3_PO_4_), which was prepared using presynthesized 2D–GeH/Ag and t‐Ag_3_PO_4_ at the optimum composition ratio. As depicted in Figure S6, the physical mixture exhibited a considerably lower TC degradation efficiency than the 2D–GeH/Ag/Ag_3_PO_4_ heterojunction (21.5% vs. 96.1% after 3 min of irradiation), supporting the benefits of the chemically assembled architecture over a simple physical mixture of the individual components.

Owing to the ultrafast degradation rates, conventional pseudo‐first‐order kinetic analysis is not informative here. Instead, charge–transfer dynamics were provided via EIS and PL. Nyquist plots (Figure 2d) investigated the photo‐excited charge transfer dynamics under open‐circuit potentials. The smallest semicircle for the 2D–GeH/Ag/Ag_3_PO_4_ heterojunction indicates the lowest interfacial charge–transfer resistance and fastest carrier transport among the tested materials. The PL spectrum of 2D–GeH/Ag/Ag_3_PO_4_ heterojunction exhibited a pronounced quenching relative to its single components under excitations resonant with each semiconductor (250 nm for t‐Ag_3_PO_4_ nanocrystals and 500 nm for pristine 2D–GeH), revealing a more efficient separation of photogenerated e^–^–h^+^ pairs in the heterojunction (Figure 2e,f) [45, 46]. These results directly correlate with the enhanced degradation and mineralization performances demonstrated by the 2D–GeH/Ag/Ag_3_PO_4_ heterojunction, making it possible to accelerate interfacial charge transfer and suppress recombination in the Z‐scheme nanoarchitecture.

Alternatively, the facet‐dependent properties of Ag_3_PO_4_ during photocatalytic degradation and mineralization of TC were investigated by synthesizing a control heterojunction containing amorphous Ag_3_PO_4_ (a‐Ag_3_PO_4_) instead of faceted t‐Ag_3_PO_4_ nanocrystals. The amorphous nature of a‐Ag_3_PO_4_ in the control 2D–GeH/Ag/a‐Ag_3_PO_4_ material was confirmed by means of TEM images (Figure S7) and XRD pattern analyses (Figure S8). As depicted in Figure S9a, the amorphous heterojunction (control 2D–GeH/Ag/a‐Ag_3_PO_4_) exhibited much lower photocatalytic efficiency than the tetrahedral‐facet 2D–GeH/Ag/Ag_3_PO_4_ heterojunction, resulting in 89.0% decomposition of TC after 3 min together with a TOC of 35.6% (Figure S9b). This demonstrates that facet engineering also plays a key role in boosting antibiotic remediation. At this point, the differences observed between degradation and mineralization efficiencies among both samples can be explained by the distinct absorption–desorption behaviors of isolated a‐Ag_3_PO_4_ and t‐Ag_3_PO_4_ toward TC molecules under dark conditions (see Figure S10, absorption efficiency at 25 min: 19% for a‐Ag_3_PO_4_ vs. 27% for t‐Ag_3_PO_4_) [39, 40]. For instance, charge separation/recombination can also be strongly affected by the crystallinity of the sample, which can be an additional factor contributing to the differences observed.

### 3.3 Rationalizing the Z‐Scheme Charge‐Transfer Mechanism

**FIGURE 3:**
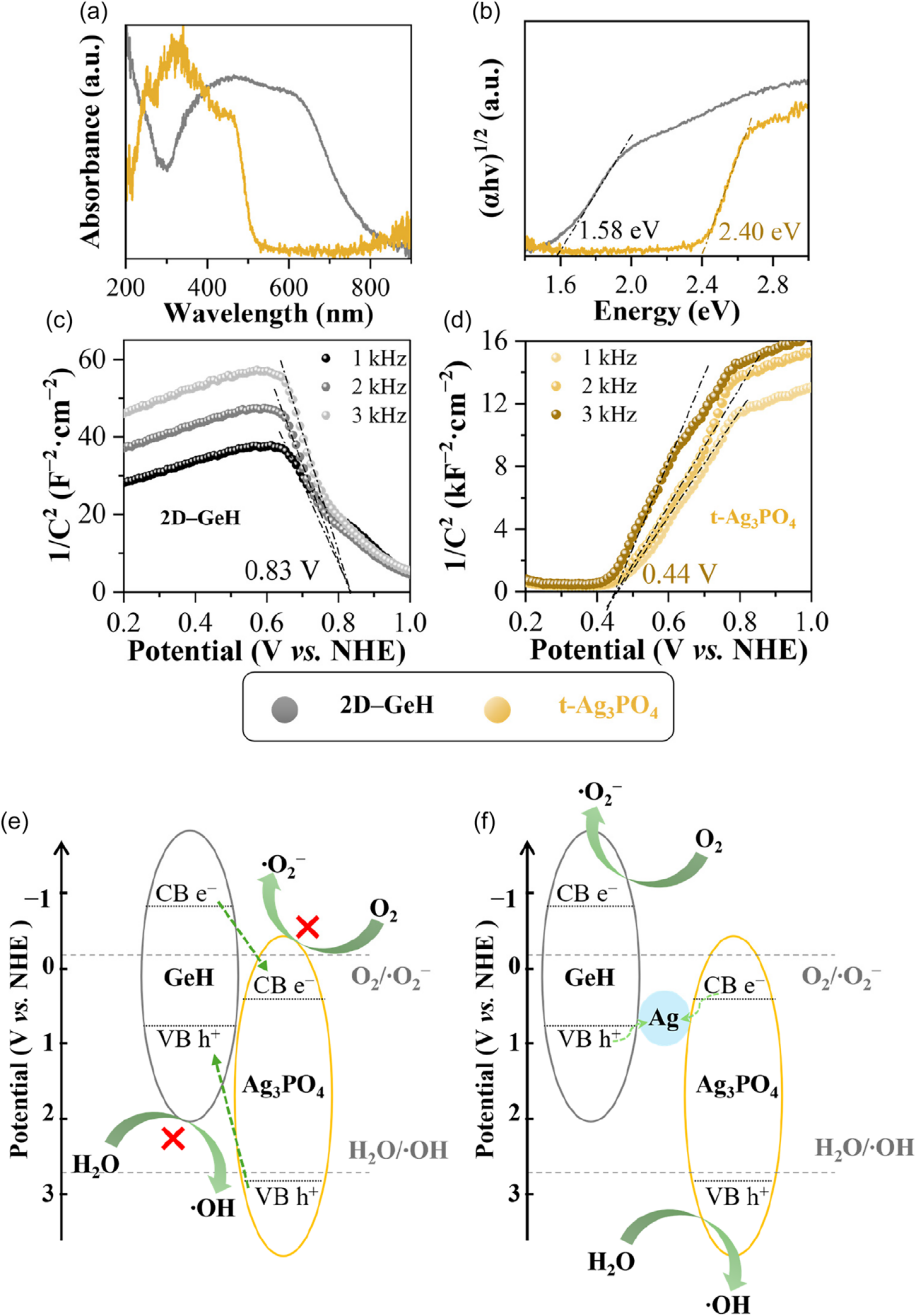
Energy band structure characterization of Z‐scheme 2D–GeH/Ag/Ag_3_PO_4_ heterojunction. (a) UV–vis spectrum of pristine 2D–GeH and t‐Ag_3_PO_4_ nanocrystals with (b) their corresponding Tauc plots. Mott–Schottky diagrams for (c) pristine 2D–GeH and (d) t‐Ag_3_PO_4_ nanocrystals. Schematic comparison of the proposed charge–transfer pathways in the 2D–GeH/Ag/Ag3PO4 heterojunction: (e) conventional type‐II heterojunction and (f) all‐solid‐state Z‐scheme heterojunction.

In order to elucidate the charge transfer pathway in the 2D–GeH/Ag/Ag_3_PO_4_ heterojunction, the band edge potentials were accurately determined via Tauc and Mott–Schottky (M–S) plots (Figure 3). On the one hand, the UV–vis spectrum for pristine 2D–GeH presented a broad absorption band at ~603 nm (Figure 3a), corresponding to an optical bandgap of ~1.58 eV according to Tauc plots (Figure 3b). This value is in concordance with our previous results [47]. On the other hand, the UV–vis spectrum for isolated t‐Ag_3_PO_4_ nanocrystals displayed an absorption band at ~500 nm, with a corresponding optical bandgap of ~2.4 eV, in line with literature [39]. The M‐S results identified pristine 2D–GeH with a p‐type semiconductor behavior (Figure 3c) with a valence band (VB) potential at 0.83 V (vs. NHE) [48, 50], while t‐Ag_3_PO_4_ behaved as an n‐type semiconductor with a conduction band (CB) at 0.44 V (Figure 3d) [51, 52].

**FIGURE 4:**
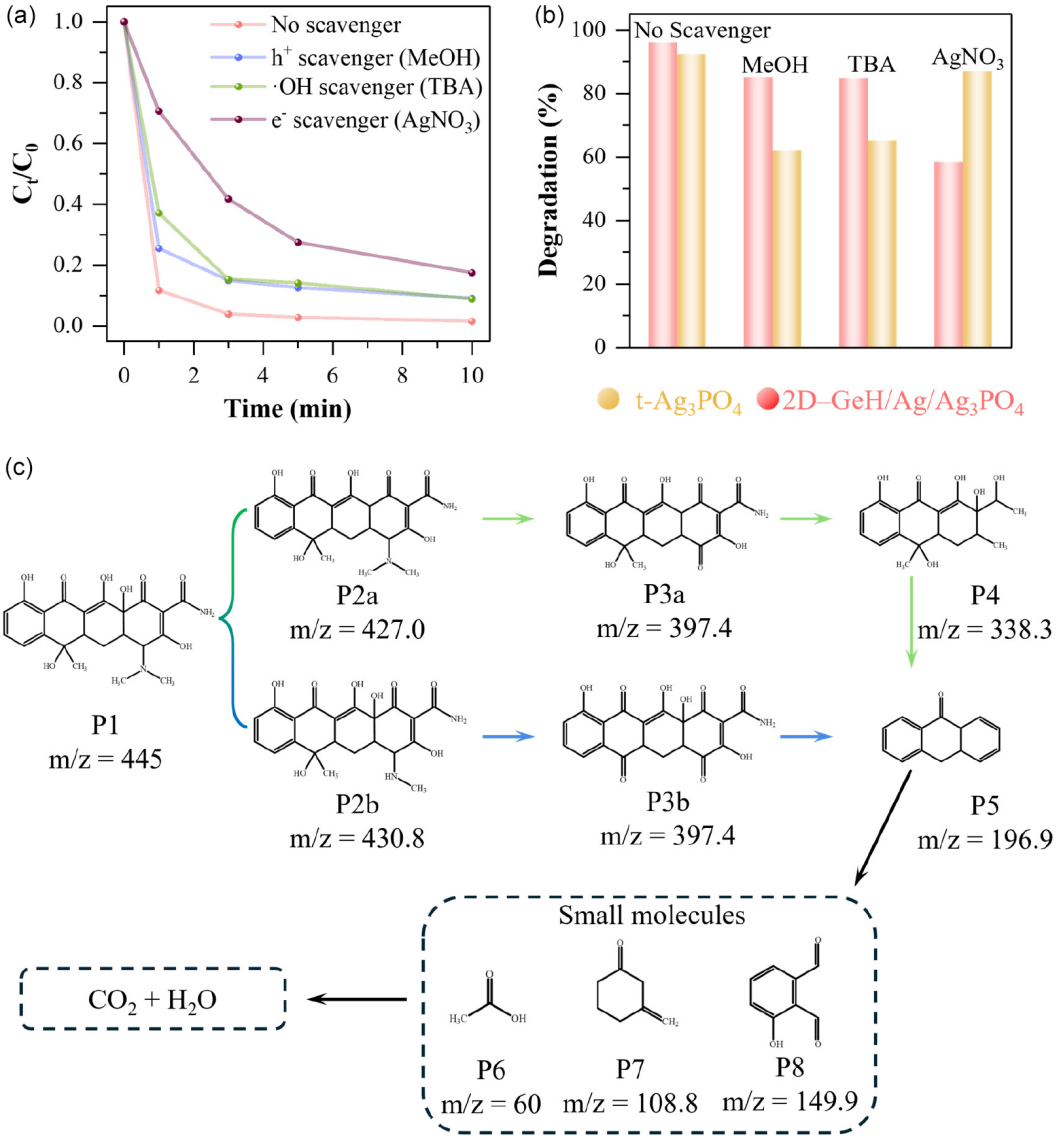
Proposed mechanism for photocatalytic process using 2D–GeH/Ag/Ag_3_PO_4_ heterojunction. Time‐dependent photocatalytic degradation of TC over (a) 2D–GeH/Ag/Ag_3_PO_4_ heterojunction and in the presences of TBA, AgNO_3_, and MeOH scavengers toward •OH, e^−^, or h^+^ active species, respectively. (b) Comparison of photocatalytic degradation efficiency between 2D–GeH/Ag/Ag_3_PO_4_ and isolated t‐Ag_3_PO_4_ with different scavengers. (c) Proposed reaction pathway for TC degradation over 2D–GeH/Ag/Ag_3_PO_4_ heterojunction.

In a conventional type‐II heterojunction, the spatial separation of charge carriers occurs at the expense of redox capability, as e^–^ transfer to the lower CB while h^+^ migrate to the higher VB. Based on the experimentally determined band‐edge positions, both type‐II (Figure 3e) and all‐solid‐state Z‐scheme (Figure 3f) charge–transfer pathways were considered for the 2D–GeH/Ag/Ag_3_PO_4_ heterojunction. While the 2D–GeH/Ag/Ag_3_PO_4_ heterojunction yielded 96.1% TC degradation, the 2D–GeH/Ag_3_PO_4_ counterpart exhibited negligible photocatalytic activity (*η* = 4.8%) in the absence of metallic Ag‐NPs (see Figure S11). This pronounced difference points out the critical role of Ag‐NPs in the charge–transfer process and supports their function as an electron mediator between 2D–GeH and t‐Ag_3_PO_4_, enabling an all‐solid‐state Z‐scheme pathway in which photogenerated e^‐^ from the CB of t‐Ag_3_PO_4_ recombine with h^+^ from the VB of 2D–GeH. As a result, the strong oxidation and reduction potentials of the remaining charge carriers are preserved, leading to enhanced photocatalytic performance. In addition, the dominant contribution of •O_2_^−^ species (Figure 4a, vide infra) is consistent with the proposed all‐solid‐state Z‐scheme mechanism depicted in Figure 3f.

To further evaluate the stability of the metallic Ag mediator during photocatalysis, the high‐resolution Ag 3d XPS spectrum was recorded before and after TC degradation (Figure S12). Only a slight increase in the Ag^0^/Ag^+^ ratio, from 0.67 to 0.78, was observed after photocatalysis, indicating that the Ag/Ag_3_PO_4_ interface remained largely preserved under the investigated reaction conditions.

### 3.4 TC Degradation Pathway

To further provide in‐depth insights into the photocatalytic activity of the 2D–GeH/Ag/Ag_3_PO_4_ heterojunction, the reaction mechanism towards TC degradation was investigated by adding different active species to the TC solution, including *t*‐butyl alcohol (TBA) [53, 54], AgNO_3_ [55, 56], and methanol (MeOH) [57, 58], which were used as scavengers for •OH, e^−^, or h^+^ species, separately. As shown in Figure 4a, all the scavengers led to significant declines in the photocatalytic degradation performance of 2D–GeH/Ag/Ag_3_PO_4_ (TC degradation: 96.1% without scavenger vs. 85.1% with MeOH, 84.6% with TBA, and 58.3% with AgNO_3_), indicating the contributions from •OH, •O_2_^–^, and h^+^ species to TC degradation. Importantly, AgNO_3_ scavenger led to the maximum decrease in photocatalytic efficiency, suggesting •O_2_^–^ as the dominant species. In contrast, control experiments using isolated t‐Ag_3_PO_4_ nanocrystals (Figure 4b) revealed a markedly different scavenger response. Significant suppression of TC degradation was observed only in the presence of TBA and MeOH, with degradation efficiencies decreasing from 92.3% to 62.1% (MeOH) and 65.0% (TBA), while a comparatively minor effect was observed with AgNO_3_ (86.9%). This behavior can be attributed to the intrinsic photocatalytic properties of t‐Ag_3_PO_4_, which efficiently generates h^+^ and •OH species with strong oxidation capabilities. However, its relatively positive conduction band potential (0.44 V vs. NHE) is insufficient to thermodynamically drive the reduction of O_2_ to •O_2_^−^ (−0.33 V vs. NHE), thereby limiting the formation of superoxide radicals via photoexcited electrons (Figure 3e). Therefore, the primary reactive species involved in TC degradation over t‐Ag_3_PO_4_ are •OH and h^+^. Besides determining the contributions of individual reactive species, these results further demonstrate the efficient utilization of both photogenerated electrons and holes in the 2D–GeH/Ag/Ag_3_PO_4_ heterojunction, thus confirming its Z‐scheme charge transfer mechanism and highlighting its superior light‐energy conversion efficiency.

The photogenerated intermediates from TC degradation under visible light irradiation were interrogated via HPLC‐MS (Figure S13). In general, the decomposition process of TC might be related to several intermediates along with different potential routes, such as demethylation, ring opening, decarbonylation, and dehydroxylation [59]. Before photocatalytic degradation, only one peak at a mass‐to‐charge ratio (*m/z*) of 445 was identified, corresponding to TC [60]. During the photocatalytic degradation process with the 2D–GeH/Ag/Ag_3_PO_4_ heterojunction, twelve intermediates were detected according to their different *m/z* fractions, which are depicted in Figure 4c. Considering the fact that the C=C, N—C, and benzene ring in TC molecules could be the most susceptible to be attacked by active oxidizing species, a TC decomposition pathway is proposed. Based on the identified intermediates, TC molecules (P1) were converted into P2a (*m/z* = 427.0) and P2b (*m/z* = 430.8, Figure S13 inset), thereby suggesting two possible transformation pathways via dehydration and demethylation, respectively [61]. According to the previous literature [61], the developmental toxicity of P2b was lower than both P1 (pristine TC) and P2a, demonstrating the advantages of the demethylation route.

Then, demethylation and carbonylation processes might occur orderly over both P2a and P2b, resulting in P3a (*m/z* = 397.4) and P3b (*m/z* = 397.4) intermediates [62]. The terminal amide‐functionalized ring of P3a would undergo ring opening via deamination and dihydroxylation reactions, yielding P4 (*m/z* = 338.3). Through continuous oxidation processes, P4 would be converted into P5 (*m/z* = 196.9) through demethylation and dihydroxylation [63]. However, no intermediate could be detected during the conversion from P3b to P5, suggesting a rapid oxidation ring‐opening reaction, which suggested the advantages of the P2b → P3b → P5 route in reaction kinetics. Finally, P5 could be decomposed into smaller intermediates, such as P6, P7, and P8 (*m/z* = 60, 108.8, and 147.9, respectively), further being mineralized into CO_2_ and H_2_O by continued oxidation and ring‐opening reactions [64, 66]. It should be noted that P5—a shared intermediate of both pathways—has been reported to show negative mutagenicity, which highlights the promising ability of the 2D–GeH‐based heterojunction in reducing mutagenic potential during photocatalytic TC degradation [63]. According to this reaction pathway, the 2D–GeH/Ag/Ag_3_PO_4_ heterojunction displays unique advantages in decomposing TC by an environmental‐friendly detoxifying route.

### 3.5 Applicability Toward Alternative Antibiotics

Finally, the broader applicability of the 2D–GeH/Ag/Ag_3_PO_4_ heterojunction toward alternative antibiotics was evaluated using ciprofloxacin (CIP), a representative fluoroquinolone antibiotic widely detected in aquatic environments [67, 68]. Experiments were conducted under the same photocatalytic conditions used for TC degradation. As shown in Figure S14, the 2D–GeH/Ag/Ag_3_PO_4_ heterojunction displayed superior photocatalytic activity toward CIP degradation (39.3%) compared with pristine 2D–GeH (5.8%) and isolated t‐Ag_3_PO_4_ (29.5%) after 10 min of visible‐light irradiation. These results confirm that the beneficial role of the heterojunction architecture is not restricted to TC degradation.

## 4 Conclusions

In this work, an all‐solid‐state Z‐scheme 2D–GeH/Ag/Ag_3_PO_4_ heterojunction was successfully synthesized via a wet‐chemical functionalization approach for efficient antibiotic degradation and mineralization, using TC as a model target. The resulting 2D–GeH/Ag/Ag_3_PO_4_ heterojunction exhibited ultrafast TC degradation (96% in 3 min) and enhanced mineralization (70.5% TOC) under visible light, also demonstrating excellent performance under simulated sunlight. The high TOC removal clearly demonstrates effective deep mineralization. Establishing a complete carbon mass balance will represent a key direction for future research to advance the mechanistic understanding of this process. Interestingly, facet engineering was found to significantly influence photocatalytic activity, particularly improving mineralization efficiency. Mechanistic investigations confirmed a Z‐scheme charge–transfer pathway mediated by Ag‐NPs, enabling efficient charge separation while preserving strong redox potentials. Reactive species analyses identified superoxide radicals as the dominant species, while HPLC‐MS revealed two main degradation routes: demethylation and dehydration. Overall, this study points out the potential of 2D–GeH‐based Z‐scheme heterojunctions as efficient AOP photocatalysts for sunlight‐driven antibiotic remediation.

## Conflicts of Interest

The authors declare no conflicts of interest.

## Supporting information

Supplementary Material

## Data Availability

The data that support the findings of this study are available from the corresponding author upon reasonable request.
