# Supplementary material for: 2D Germanane‐Based Z‐Scheme Heterojunctions for Ultrafast Antibiotic Degradation and Mineralization
Source: Small Sci. 2026 Aug 19;6(8):e70375. doi: 10.1002/smsc.70375 (PMC13489318; doi:10.1002/smsc.70375)
Supplement: Supplementary file 1 [file SMSC-6-e70375-s001.pdf]

2D Germanane-based Z-Scheme Heterojunctions for Ultrafast Antibiotic Degradation and Mineralization

*Yiming Lei,[a] Xavier Sala,[a] Jordi García-Antón,[a] and Jose Muñoz[a],**

Supporting information

Supporting Figures

Figure S1. TEM images of t-Ag_3PO_4 ……………………………………………………….. **S2**

Figure S2. UV-vis spectrum of TC without and with 2D–GeH/Ag/t-Ag_3PO_4 ………………. **S2**

Figure S3. Kinetic analysis ………………………………………………………………. **S3**

Figure S4. Stability test ………………………………………………………………….. **S3**

Figure S5. HR-XPS of Ag 3d before and after photocatalytic reaction ……………………. **S4**

Figure S6. Photocatalytic degradation over Mix 2D–GeH/Ag/Ag_3PO_4 …………………… **S4**

Figure S7. Photocatalytic degradation under visible and solar light ……………………….. **S5**

Figure S8. TEM images of a-Ag_3PO_4 and 2D–GeH/Ag/a-Ag_3PO_4 …………..……………… **S5**

Figure S9. XRD patterns of pristine 2D-GeH, a-Ag_3PO_4, and 2D–GeH/Ag/a-Ag_3PO_4 ……. **S6**

Figure S10. Facet dependence on photocatalytic degradation ….…………………………... **S6**

Figure S11. Adsorption-desorption equilibrium …….…………..……..…………………… **S7**

Figure S12. Photocatalytic degradation of 2D–GeH/Ag/Ag_3PO_4 and 2D–GeH/Ag_3PO_4 …… **S7**

Figure S13. HPLC-MS spectra of photocatalytic TC degradation …..……..…………….…. **S8**

Figure S14. Photocatalytic CIP degradation …..……..……..……..……..……..……..……. **S8**

Supporting Tables

Table S1. Percentage of each element in the 2D–GeH/Ag/Ag_3PO_4 heterojunction ……….. **S9**

Table S2. Photocatalytic efficiency of 2D–GeH-based sample toward TC degradation …… **S9**

Table S3. Comparison table of different photocatalysts toward TC degradation …………… **S10**

References

Supporting Figures

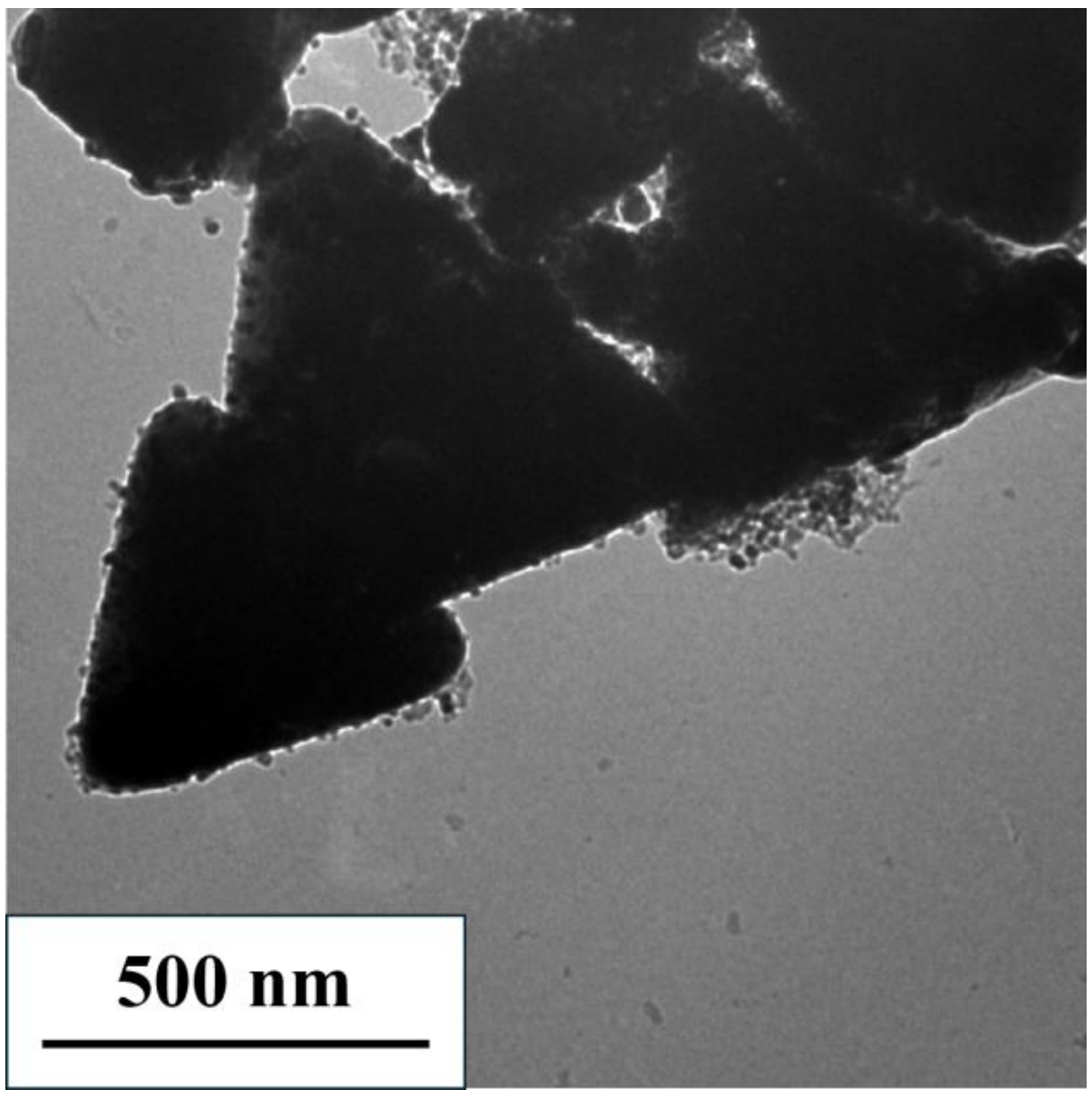

Fig. S1. TEM images of isolated t-Ag_3PO_4.

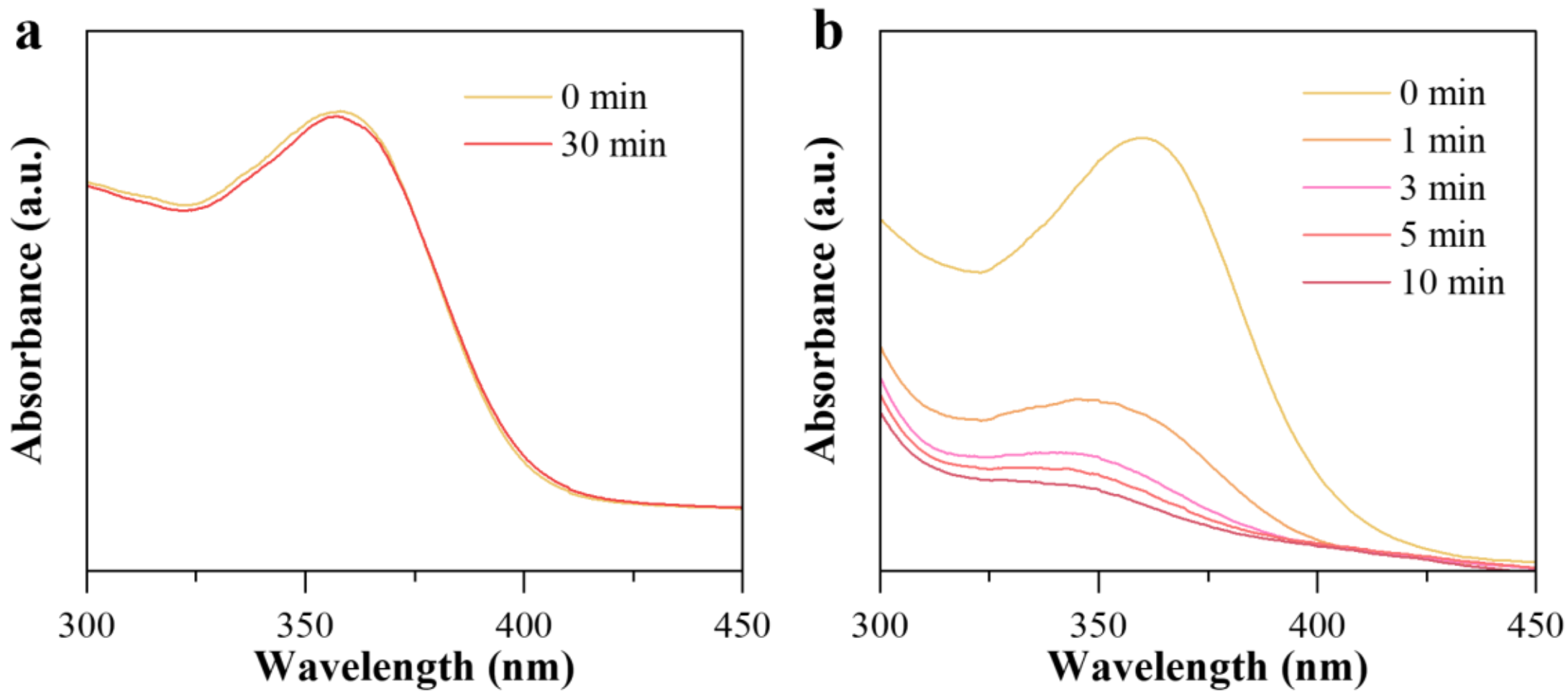

Fig. S2. UV-vis spectrum of 20 mg·L^{-1} TC **a)** in the absence **b)** and the presence of 2D–GeH/Ag/Ag_3PO_4 heterojunction at different irradiation times.

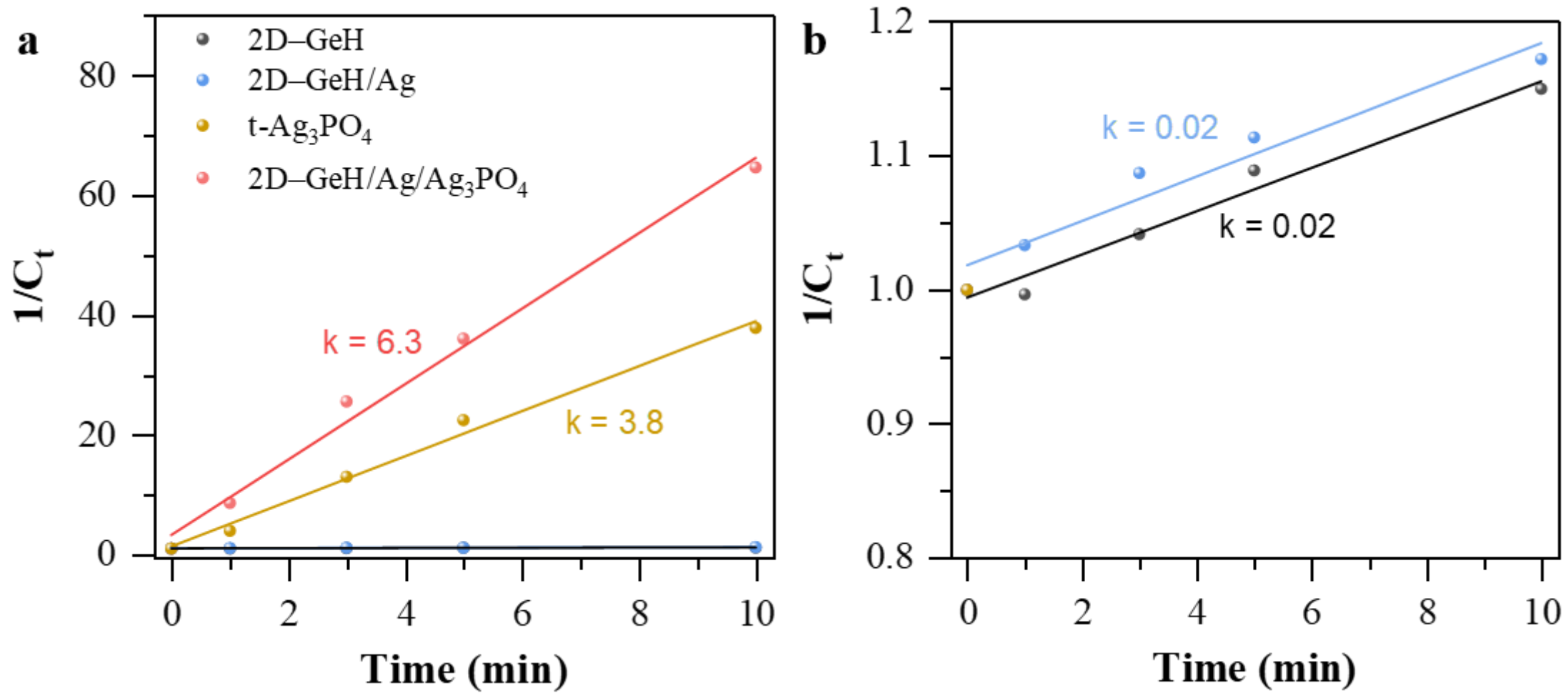

Fig. S3. a) TC degradation kinetic curves over pristine 2D–GeH, 2D–GeH/Ag, Ag_3PO_4, and 2D–GeH/Ag/Ag_3PO_4 heterojunction. **b)** Partial enlarged detail of the low-kinetics region.

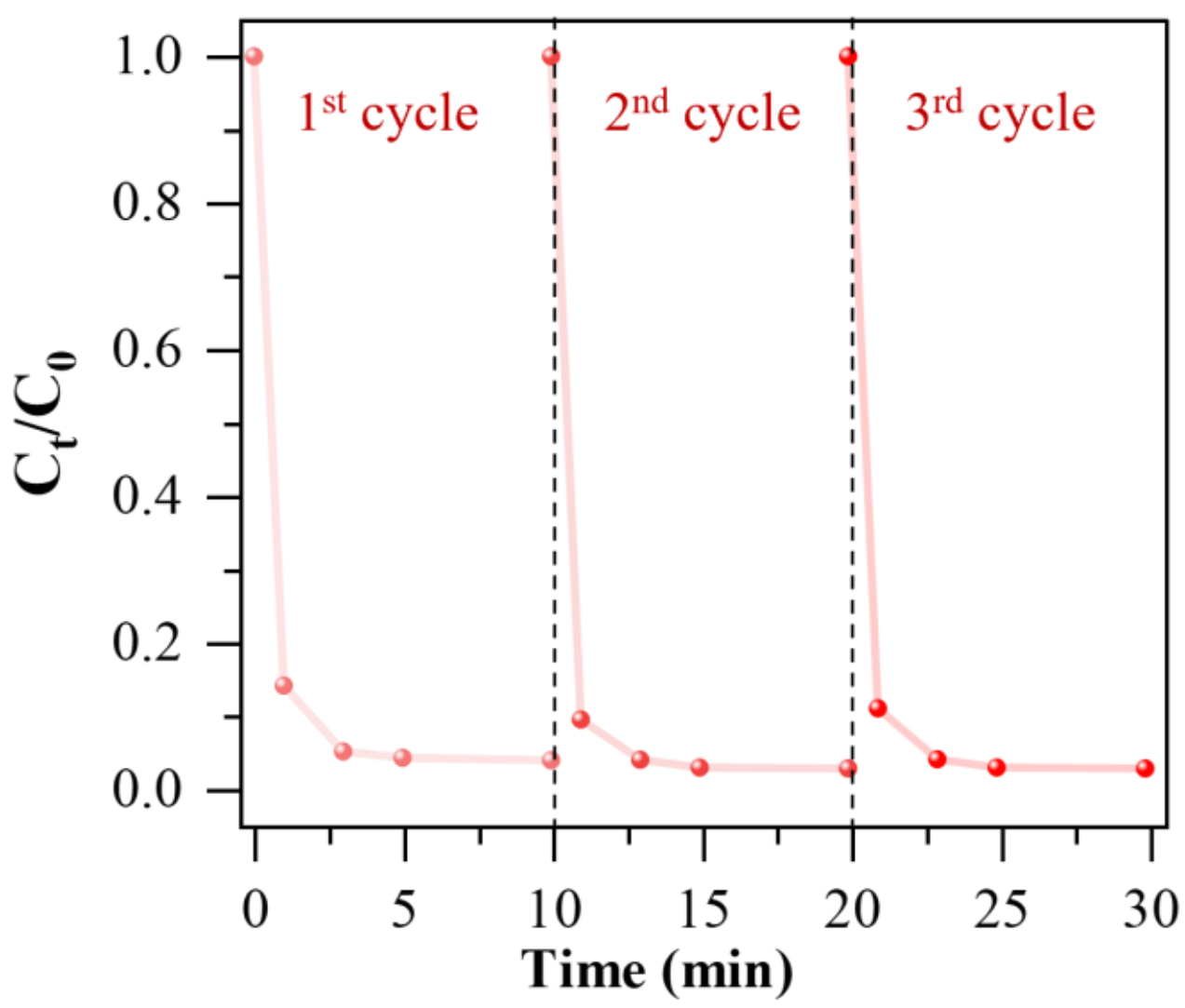

Fig. S4. Cycling stability test of photocatalytic degradation performance over 2D–GeH/Ag/Ag_3PO_4 heterojunction. Reaction conditions: initial TC concentration: 20 mg· L^{-1}; material dispersion: 0.2 mg· mL^{-1}, light source: LED lamp with visible light (wavelength: 420–780 nm; light intensity: 1 sun).

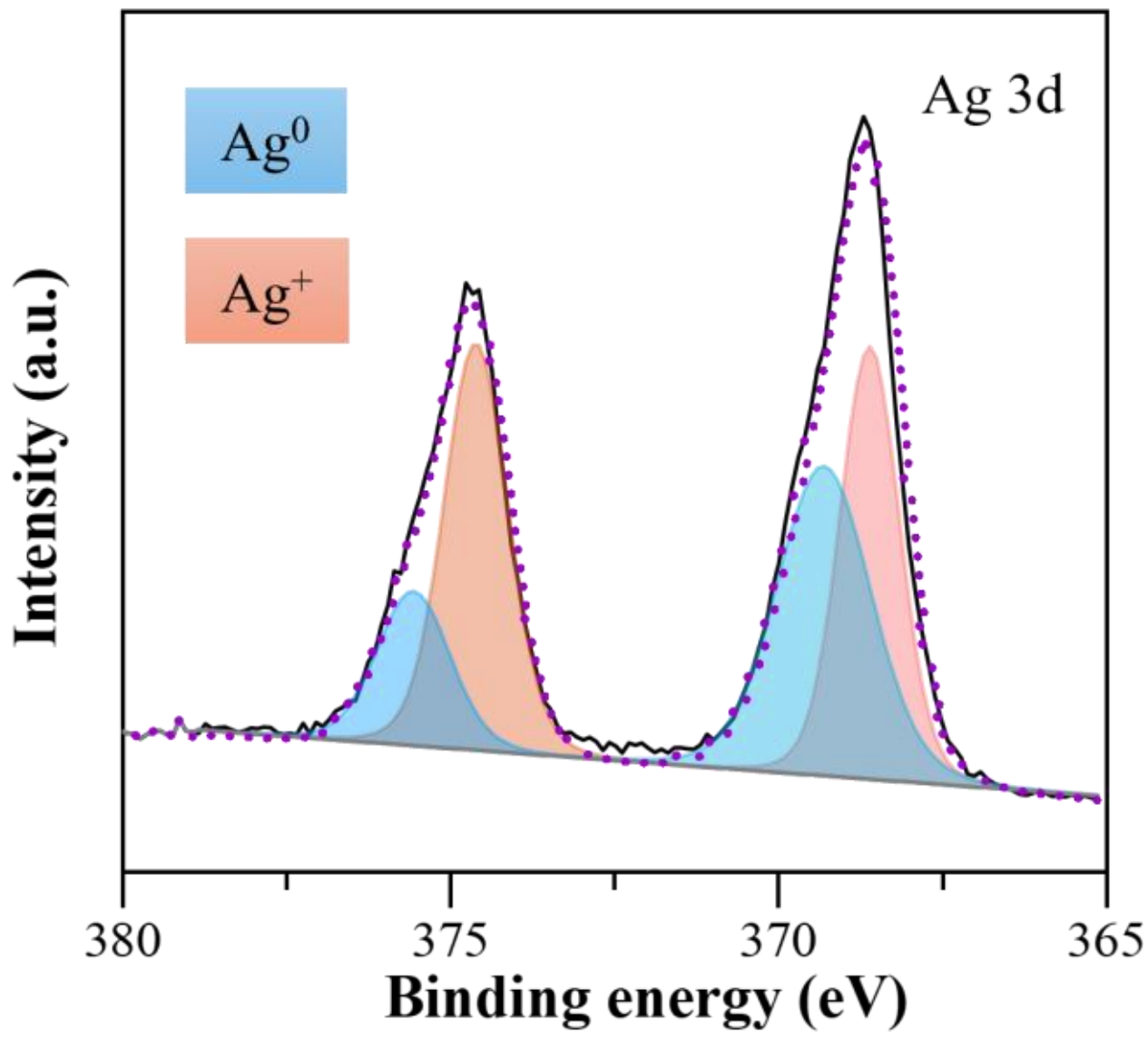

Fig. S5. High-resolution XPS spectrum of the Ag 3d core-level for 2D–GeH/Ag/Ag_3PO_4 heterojunction before and after TC degradation experiments

.

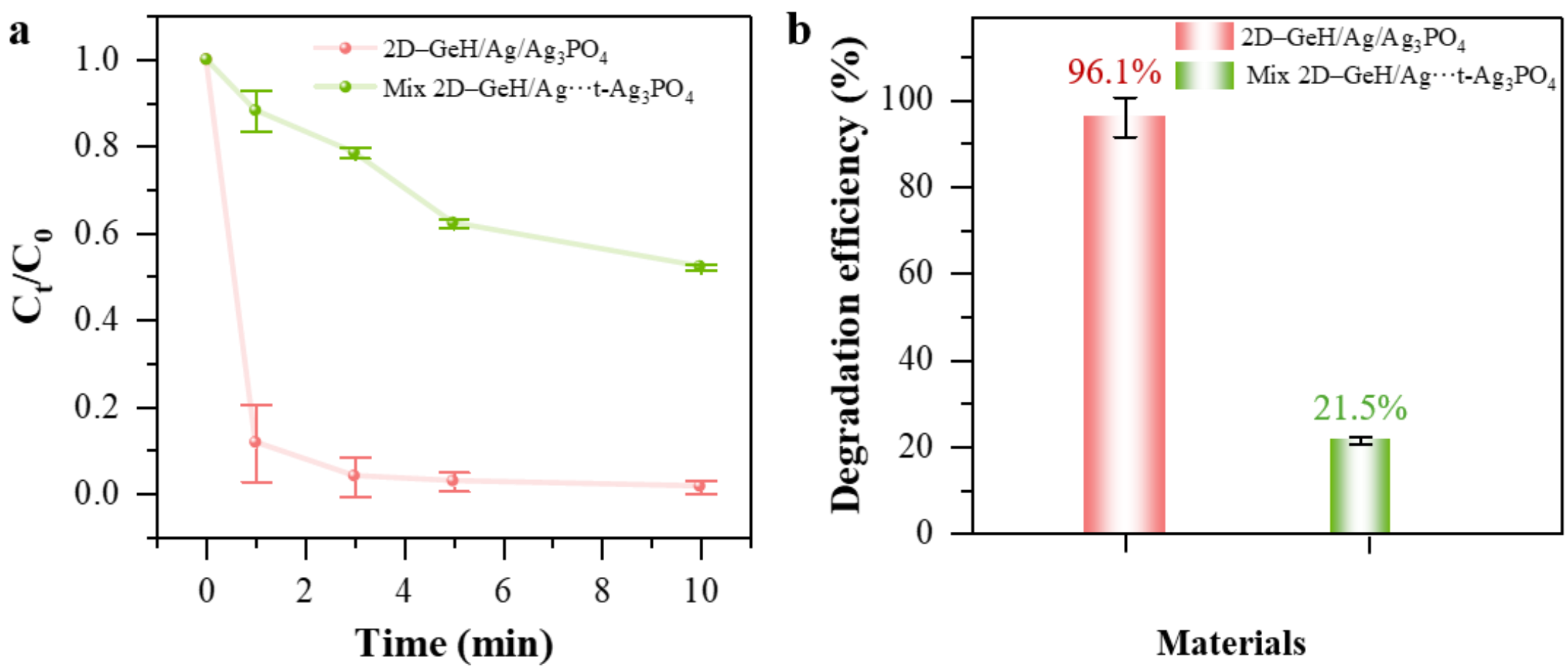

Fig S6. a) Time-dependent photocatalytic degradation of TC over 2D–GeH/Ag/Ag_3PO_4 heterojunction and Mix 2D–GeH/Ag/Ag_3PO_4. **b)** Comparison in CIP degradation efficiency of 2D–GeH/Ag/Ag_3PO_4 heterojunction and Mix 2D–GeH/Ag/Ag_3PO_4 after irradiation by visible light for 3 min. Reaction conditions: initial TC concentration: 20 mg· L^{-1}; material dispersion: 0.2 mg· mL^{-1}, light source: LED lamp with visible light (wavelength: 420–780 nm; light intensity: 1 sun).

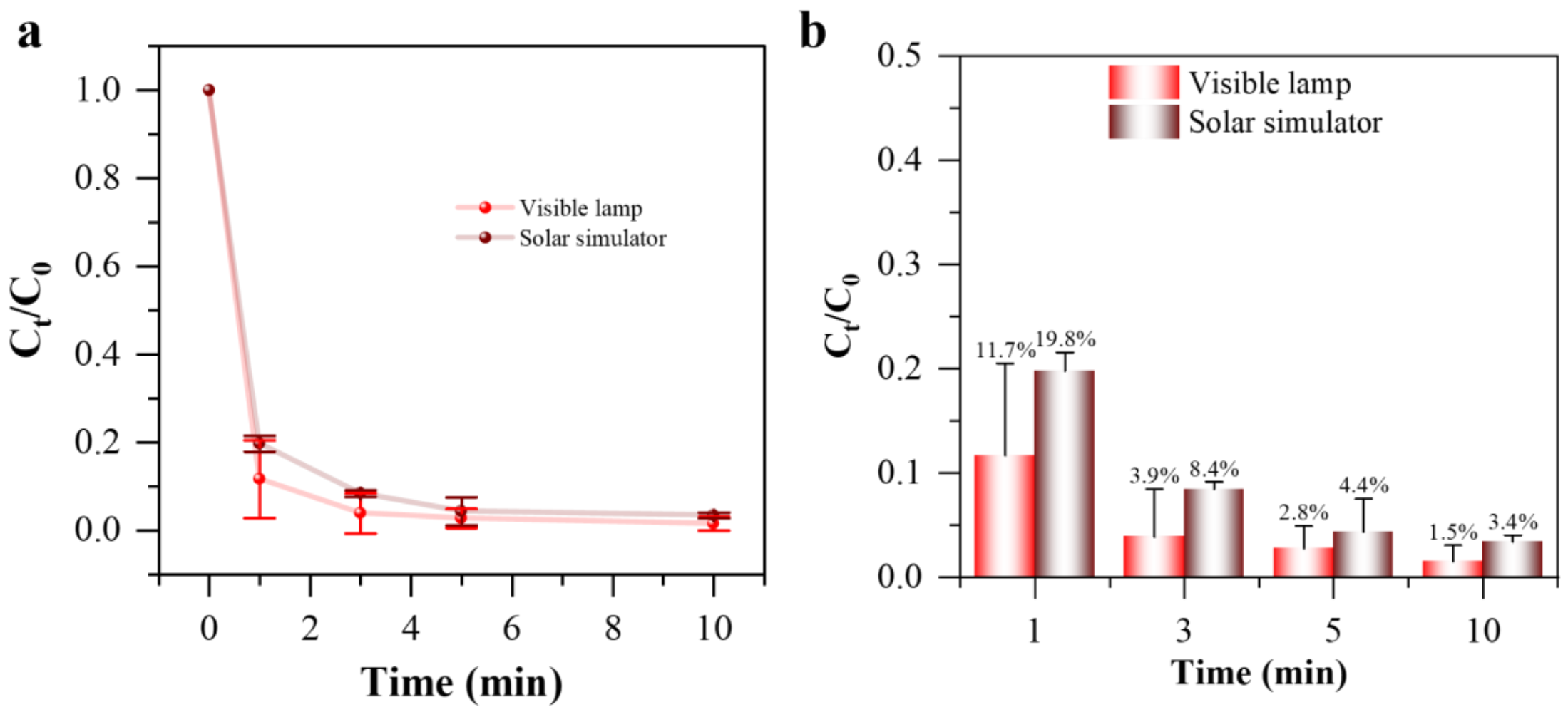

Fig. S7. a) Time-dependent photocatalytic degradation of TC over 2D–GeH/Ag/Ag_3PO_4 under visible light (light source: LED lamp; light intensity: 1 sun) and solar light (light source: solar simulator; light intensity: 1 sun) irradiation conditions, and **b)** corresponding comparison histogram of TC residual amount.

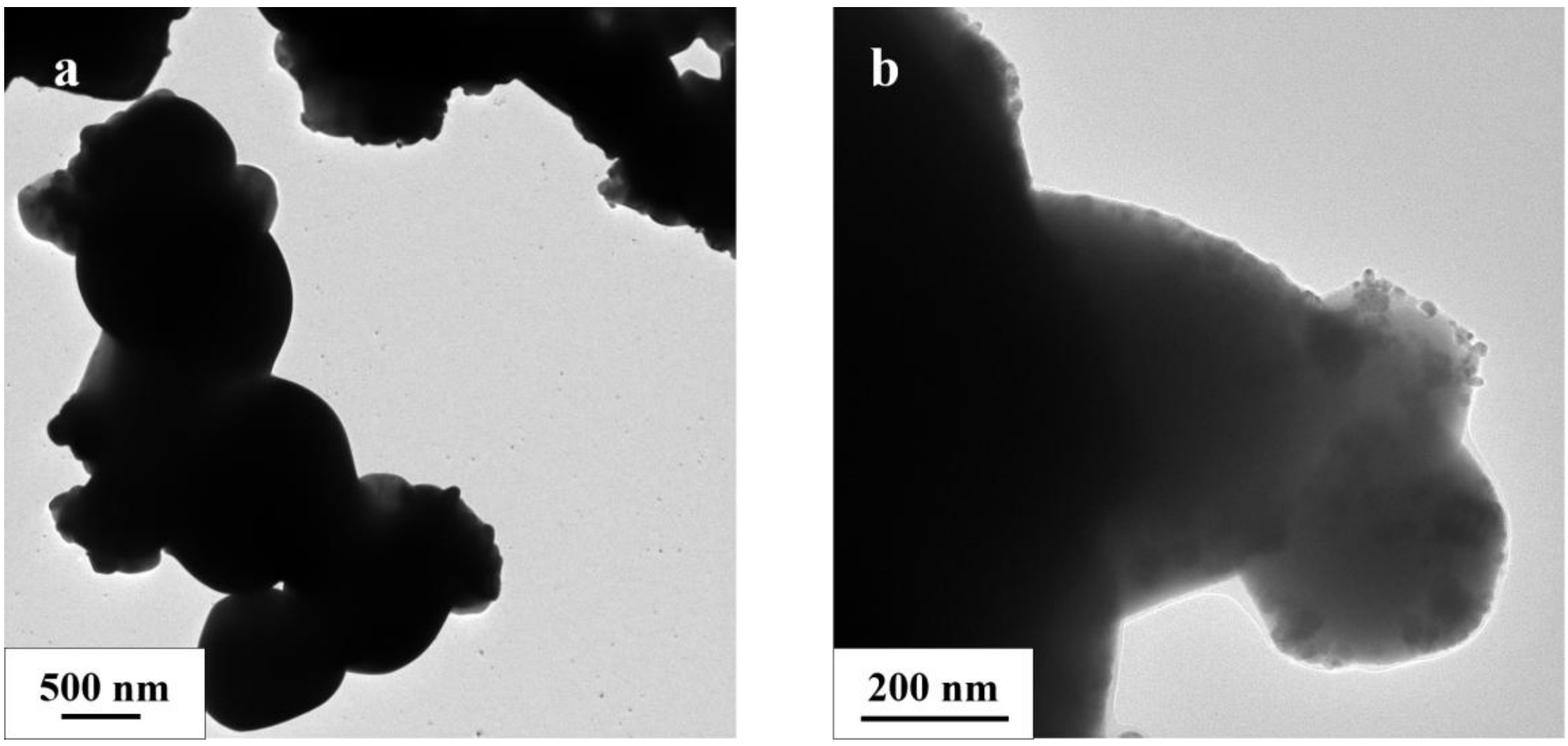

Fig. S8. TEM images of **a)** isolated a-Ag_3PO_4 and **b)** control 2D–GeH/Ag/a-Ag_3PO_4 material.

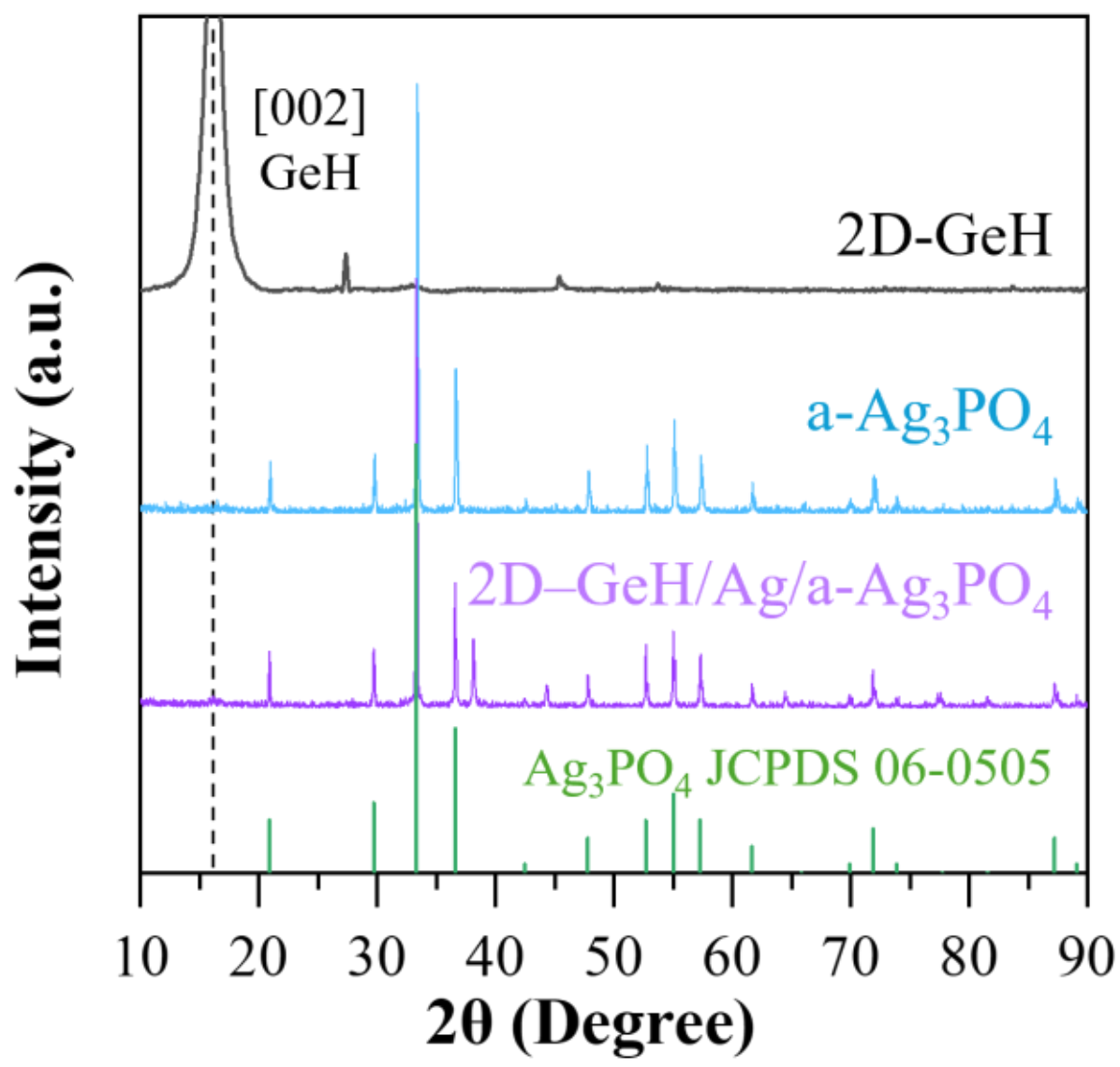

Fig. S9. XRD patterns of pristine 2D-GeH, a-Ag_3PO_4, and control 2D–GeH/Ag/a-Ag_3PO_4 material.

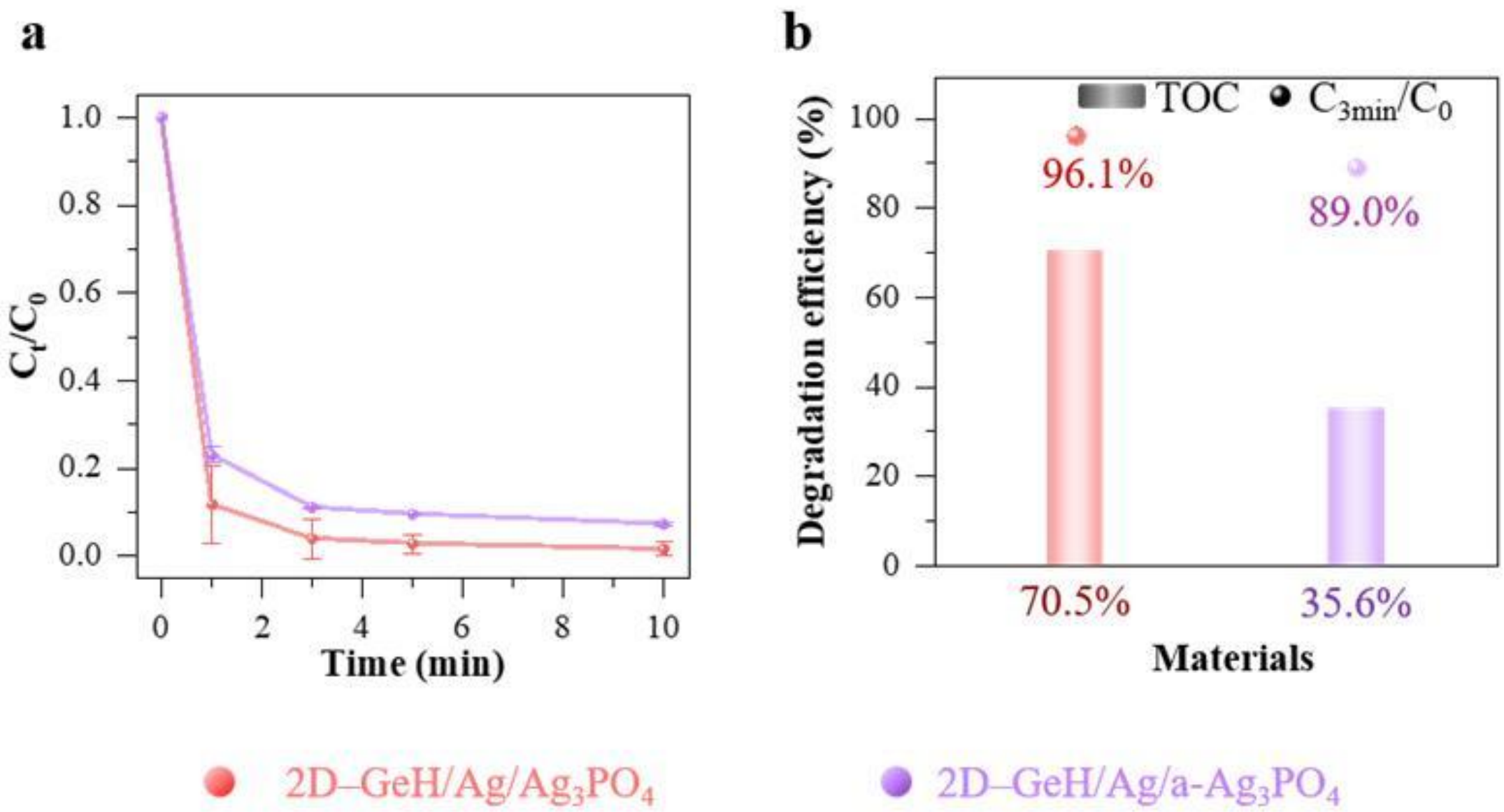

Fig. S10. Time-dependent photocatalytic degradation of TC over control 2D–GeH/Ag/a-Ag_3PO_4 and tetrahedral-facet 2D–GeH/Ag/Ag_3PO_4 heterojunction (labeled here as 2D–GeH/Ag/t-Ag_3PO_4 for proper comparison).

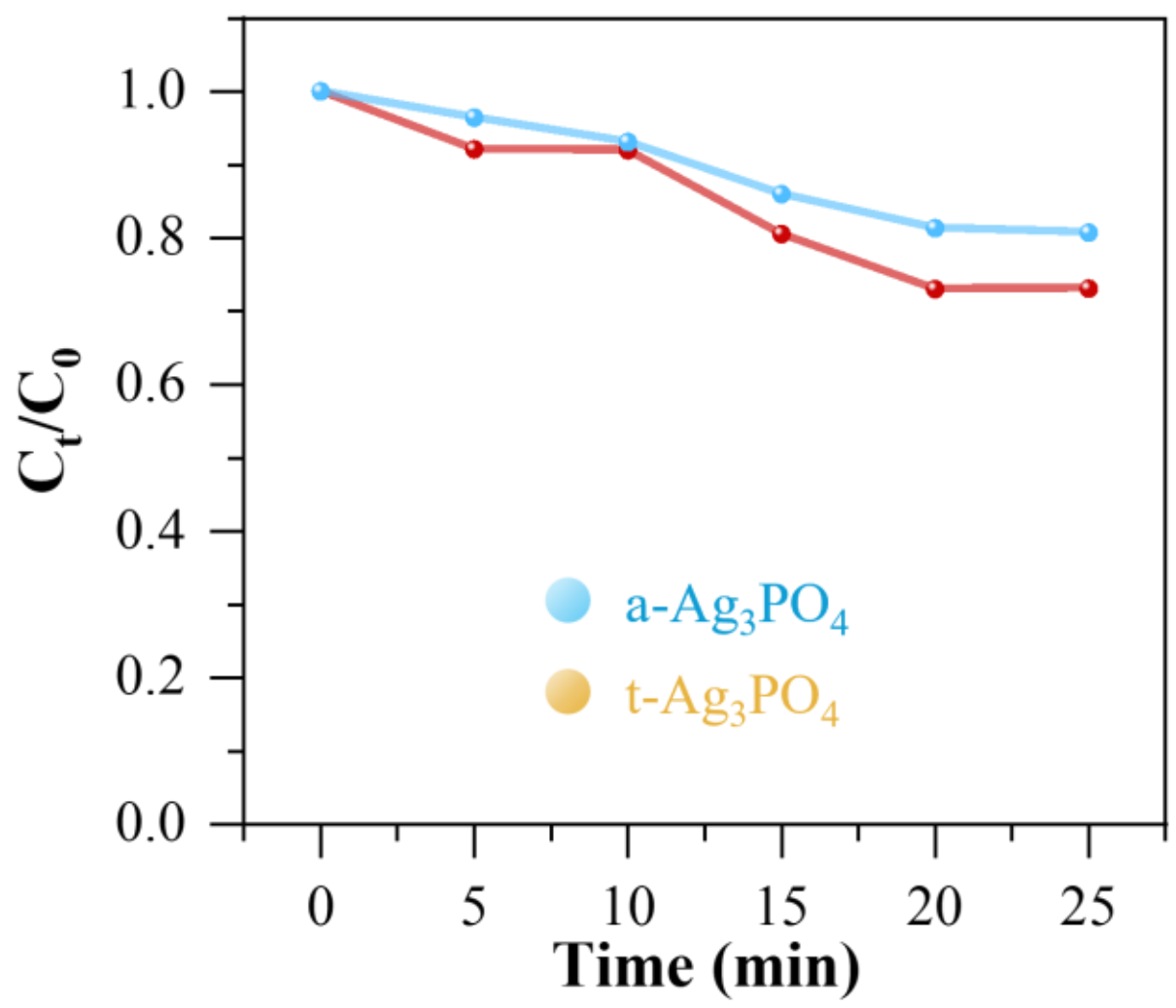

Fig. S11. Adsorption-desorption equilibrium of pristine 2D−GeH, t-Ag_3PO_4, and a-Ag_3PO_4 towards TC under dark conditions.

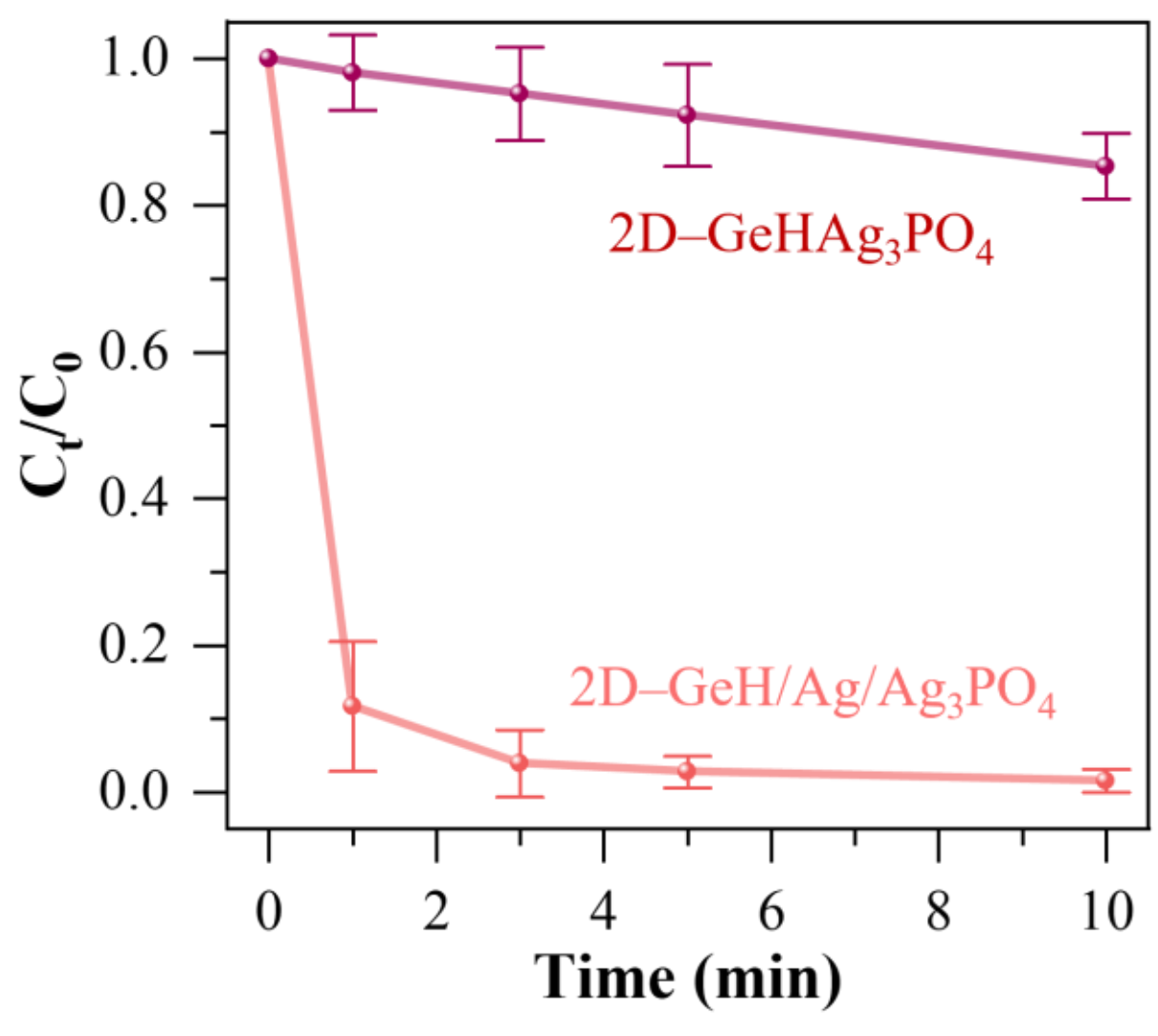

Fig. S12. Time-dependent photocatalytic degradation of TC over 2D–GeH/Ag/Ag_3PO_4 and 2D–GeH/Ag_3PO_4. Reaction conditions: initial TC concentration: 20 mg· L^{-1}; material dispersion: 0.2 mg· mL^{-1}, light source: LED lamp with visible light (wavelength: 420–780 nm; light intensity: 1 sun).

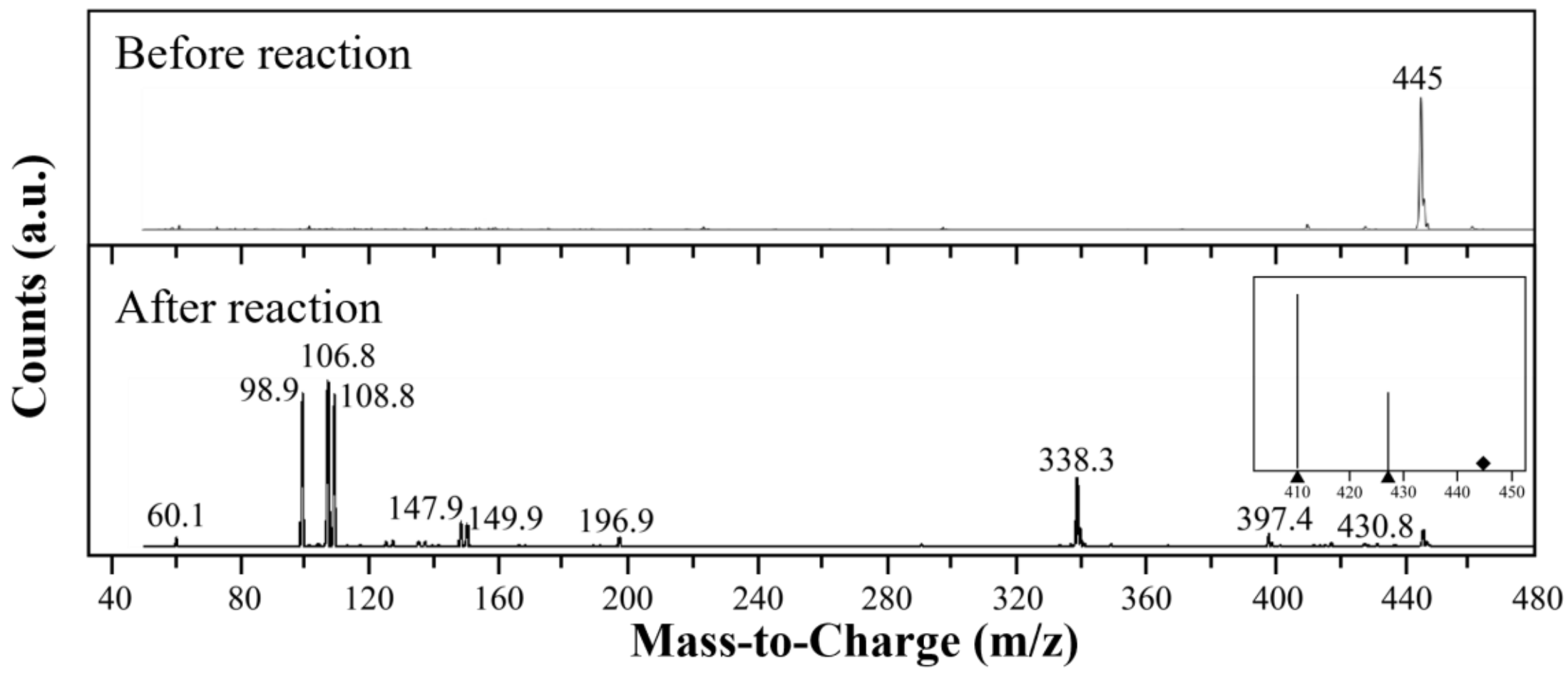

Fig. S13. HPLC-MS spectra of TC degradation before and after photocatalytic reaction. Inset: MS2 analysis for the peak at 445 m/z.

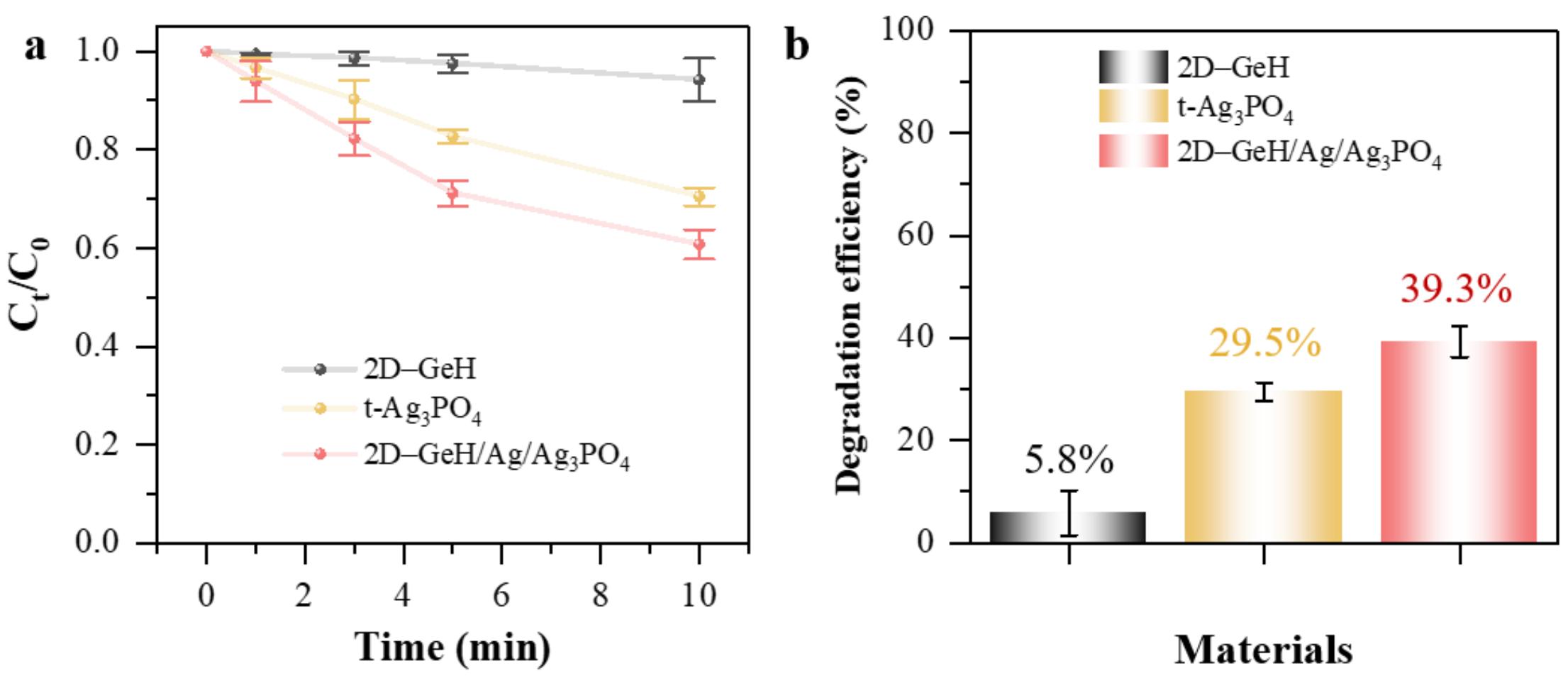

Fig. S14. a) Time-dependent photocatalytic degradation of ciprofloxacin (CIP) over pristine 2D-GeH, t-Ag_3PO_4, and 2D–GeH/Ag/Ag_3PO_4 heterojunction. **b)** Comparison in CIP degradation efficiency of pristine 2D-GeH, t-Ag_3PO_4, and 2D–GeH/Ag/Ag_3PO_4 heterojunction after irradiation by visible light for 10 min. Reaction conditions: initial CIP concentration: 10 mg· L^{-1}; material dispersion: 0.2 mg· mL^{-1}, light source: LED lamp with visible light (wavelength: 420–780 nm; light intensity: 1 sun).

Supporting Tables

Table S1. Percentage of each element in the 2D–GeH/Ag/Ag_3PO_4 heterojunction.

Element	Percentage
**Ge**	16.9%
**Ag**	10.6% (Ag^0)<br>15.9% (Ag^+)
**O**	40.8%
**P**	15.8%

Table S2. Comparison of the photocatalytic efficiency of the 2D–GeH/Ag/Ag_3PO_4 heterojunction with other recently reported photocatalysts for TC degradation (initial TC concentration: 20 mg· L^{-1}).

Photocatalysts	TC degradation efficiency	Time (min)	Irradiation source	Ref.
**2D–GeH/Ag/t-Ag_3PO_4**	96.1%	3	Visible light (λ : 420 – 800 nm)	This work
**In-Nv-CNF**	94%	30	Xe lamp (λ > 420 nm)	Adv. Mater. 2024[1]
**$LaFeO_3$@PE**	~ 80%	120	Visible light	Photochem. Photobiol., A 2021[2]
**NH_2-MIL-125(Ti)**	~ 90%	180	Xe lamp (λ > 320 nm)	J. Environ. Chem. Eng. 2021[3]
**SAC Ag-g-C_3N_4**	91.1%	60	Xe lamp (λ > 420 nm)	Water Res. 2021[4]
**SAC Ag/mpg-C_3N_4**	43%	60	Xe lamp (λ > 400 nm)	Appl. Catal., B 2017[5]
**SAC Mo^1@CNNTs**	97.3%	60	Xe lamp (λ > 420 nm)	Chem. Eng. J. 2023[6]
**Multi-layered CNIS**	82.6%	10	Visible light	Chem. Eng. J. 2023[7]
**g-C_3N_4/ LDH**	95%	90	Xe lamp (λ > 420 nm)	Sep. Purif. Technol. 2023[8]
**BiOBr-Bi_2WO_6**	96%	120	Xe lamp (λ > 420 nm)	Sep. Purif. Technol. 2021[9]
**3D CN foam**	64.2%	70	Xe lamp (λ > 420 nm)	Water Res. 2018[10]

Table S3. Photocatalytic performance of control materials (*viz.* pristine 2D–GeH, 2D–GeH/Ag, 2D–GeH/Ag_3PO_4, and isolated t-Ag_3PO_4) compared with the 2D–GeH/Ag/Ag_3PO_4 heterojunction for TC degradation (initial TC concentration: 20 mg· L^{-1}).

Photocatalyst	TC degradation efficiency
**2D–GeH/Ag/t-Ag_3PO_4**	96.1%
**pristine 2D–GeH**	4.0%
**2D–GeH/Ag**	8.0%
**2D–GeH/Ag_3PO_4**	4.8%
**isolated t-Ag_3PO_4**	92.3%

References

[1] J. Zhang, X. Yang, G. Xu, B. K. Biswal, R. Balasubramanian, *Adv. Mater.* **2024**, *36*, 2309205.

[2] Ö. Tuna, E. B. Simsek, *J. Photochem. Photobiol. A Chem.* **2021**, *418*, 113405.

[3] R. R. Solís, A. Gómez-Avilés, C. Belver, J. J. Rodriguez, J. Bedia, *J. Environ. Chem. Eng.* **2021**, *9*, 106230.

[4] Z. Zhou, Z. Shen, C. Song, M. Li, H. Li, S. Zhan, *Water Res.* **2021**, *201*, 117314.

[5] Y. Wang, X. Zhao, D. Cao, Y. Wang, Y. Zhu, *Appl. Catal. B Environ.* **2017**, *211*, 79.

[6] M. Mateen, W.-C. Cheong, C. Zheng, S. H. Talib, J. Zhang, X. Zhang, S. Liu, C. Chen, Y. Li, *Chem. Eng. J.* **2023**, *451*, 138305.

[7] J. Zhang, R. Balasubramanian, X. Yang, *Chem. Eng. J.* **2023**, *453*, 139776.

[8] J. Zheng, C. Fan, X. Li, Q. Yang, D. Wang, A. Duan, S. Pan, B. Zhang, J. Ding, S. Rong, H. Yin, *Sep. Purif. Technol.* **2023**, *305*, 122554.

[9] K. Liu, H. Zhang, Y. Muhammad, T. Fu, R. Tang, Z. Tong, Y. Wang, *Sep. Purif. Technol.* **2021**, *274*, 118992.

[10] H. Wang, Y. Wu, M. Feng, W. Tu, T. Xiao, T. Xiong, H. Ang, X. Yuan, J. W. Chew, *Water Res.* **2018**, *144*, 215.
